# Cardiovascular risk assessment methods yield unequal risk predictions: a large cross-sectional study in psychiatric secondary care outpatients

**DOI:** 10.1186/s12888-023-05022-1

**Published:** 2023-07-24

**Authors:** Davy Quadackers, Edith Liemburg, Fionneke Bos, Bennard Doornbos, Arne Risselada, PHAMOUS investigators, Agna Bartels-Velthuis, Richard Bruggeman, Stynke Castelein, Frederike Jörg, Henderikus Knegtering, Marieke Pijnenborg, Marjolein Berger, Ellen Visser, Danielle Cath

**Affiliations:** 1 Mental Health Services Drenthe, P.O. box 30007, 9400 RA Assen, The Netherlands; 2 Department of Psychiatry, University of Groningen, University Medical Center Groningen, Rob Giel Research Center, P.O. box 30.001, 9700 RB Groningen, The Netherlands; 3 Department of Clinical Psychology & Experimental Psychopathology, University of Groningen, Faculty of Behavioural and Social Sciences, Grote Kruisstraat 2/1, 9712 TS Groningen, The Netherlands; 4 Research Department, Lentis Psychiatric Institute, Hereweg 80, 9725 AG Groningen, The Netherlands; 5 Department of Clinical Pharmacy, Wilhelmina Hospital, Assen, The Netherlands; 6 Department of General Practice & Elderly Care Medicine, University of Groningen, University Medical Center Groningen, Groningen, the Netherlands

**Keywords:** Cardiovascular risk assessment, Psychiatric patients, Metabolic syndrome

## Abstract

**Background:**

Patients with a mental illness are more likely to develop, and die from, cardiovascular diseases (CVD), necessitating optimal CVD-risk (CVR)-assessment to enable early detection and treatment. Whereas psychiatrists use the metabolic syndrome (MetS)-concept to estimate CVR, GPs use absolute risk-models. Additionally, two PRIMROSE-models have been specifically designed for patients with severe mental illness. We aimed to assess the agreement in risk-outcomes between these CVR-methods.

**Methods:**

To compare risk-outcomes across the various CVR-methods, we used somatic information of psychiatric outpatients from the PHAMOUS-, and MOPHAR-database, aged 40–70 years, free of past or current CVD and diabetes. We investigated: (1) the degree-of-agreement between categorical assessments (i.e. MetS-status vs. binary risk-categories); (2) non-parametric correlations between the number of MetS-criteria and absolute risks; and (3) strength-of-agreement between absolute risks.

**Results:**

Seven thousand twenty-nine measurements of 3509 PHAMOUS-patients, and 748 measurements of 748 MOPHAR-patients, were included. There was systematic disagreement between the categorical CVR-assessments (all *p* < 0.036). Only MetS-status versus binary Framingham-assessment had a fair strength-of-agreement (*κ* = 0.23–0.28). The number of MetS-criteria and Framingham-scores, as well as MetS-criteria and PRIMROSE lipid-scores, showed a moderate-strong correlation (*τ* = 0.25–0.34). Finally, only the continuous PRIMROSE desk and lipid-outcomes showed moderate strength-of-agreement (*ρ* = 0.91).

**Conclusions:**

The varying methods for CVR-assessment yield unequal risk predictions, and, consequently, carry the risk of significant disparities regarding treatment initiation in psychiatric patients. Considering the significantly increased health-risks in psychiatric patients, CVR-models should be recalibrated to the psychiatric population from adolescence onwards, and uniformly implemented by health care providers.

**Trial registration:**

The MOPHAR research has been prospectively registered with the Netherlands Trial Register on 19th of November 2014 (NL4779).

**Supplementary Information:**

The online version contains supplementary material available at 10.1186/s12888-023-05022-1.

## Background

Across a wide range of psychiatric diagnoses, patients have a life expectancy that is reduced by 10–20 years compared to the general population [1]. Various explanations have been proposed for this health disparity [2]. First, compared to their healthy peers, patients with psychotic disorders, major depression, bipolar disorder and anxiety disorders may have differential and mostly increased exposure to risk factors, such as an unhealthy lifestyle (e.g., smoking, substance use disorders including alcohol, unhealthy diets and less physical activity [3–8]). Psychiatric patients are more likely to develop cardiovascular diseases (CVD), and their CVD-related mortality risk is doubled to tripled [9, 10]. Second, increased mortality may be due to iatrogenic effects of psychopharmacological treatment, including weight gain [11]. Third, patients may experience inequity in and access to health care services [2]. Other risk-increasing factors are sympathetic overactivity, genetic polymorphisms interacting with cardiovascular risk factors, inflammation, and platelet dysfunction [12]. Finally, mere presence of psychiatric disease has been demonstrated to independently contribute to CVD-risk [13]. All of these established risk factors are known to interact synergistically and increase the likelihood to develop CVD, especially when multiple factors co-occur [14, 15].

Given the high mortality risk, it is widely acknowledged that somatic health screening should routinely be offered to patients with psychiatric disorders [16]. However, the physical health status of psychiatric patients is not always routinely monitored [17], and when monitored, deficits are not systematically treated [18]. Furthermore, in the Netherlands, as in the vast majority of other countries, there is a lack of clarity and consensus about which professional should be responsible for detecting and managing physical problems in these patients: the treating psychiatrist or the general practitioner? Fragmented medical and mental health care systems, and lack of integrated services, could be additional factors that contribute to the increasing health gap between psychiatric and non-psychiatric patients [17, 19].

Generally, in psychiatric care, different approaches are used compared to general practice to assess cardiovascular risks, which can lead to divergent predictions of CVD-morbidity and -mortality, depending on the service the patient attends. Subsequently, patients with a psychiatric diagnosis may receive, or not, varying (prophylactic) somatic treatments depending on whether their psychiatrist takes care of the primary prevention of CVD, as opposed to their GP.

Psychiatrists, on the one hand, generally use the presence or absence of the metabolic syndrome (MetS) to assess cardiovascular risk [20]. MetS entails a cluster of factors identifying patients at increased cardiometabolic risk [21]. Individuals with MetS are at increased risk for developing CVD, and are three times more likely to die hereof [22]. Psychiatric patients are especially vulnerable, as illustrated by the MetS prevalence being 58% higher in this group compared to the general population [12, 22]. The presence of MetS predicts CVD events with an overall accuracy of 63% (95% CI area under the receiver-operating characteristic curve (AUC): 56%–69%; [23]). An AUC of 0% means that the model wrongly predicts the outcome in every instance, whereas an AUC of 100% indicates perfect prediction. Nonetheless, some experts dispute the diagnostic, prognostic, and therapeutic value of the MetS concept. A first criticism entails the dichotomous nature (i.e. presence/absence) of the concept, and consequently its relative insensitivity to subtle early warning signs of CVD [24, 25]. Overall, patients are monitored on its presence or absence, thus having the risk of being treated only when MetS is present, leaving people who display one or two risk factors sometimes untreated. Another point of criticism is that important additional cardiovascular determinants, such as age, sex, and smoking status, are not taken into account in the MetS-system.

GPs, on the other hand, generally use continuous -and thus more flexible- scoring systems, such as the Framingham [26], and ‘Systematic Coronary Risk Evaluation’ (SCORE)-model [27], the latter being used in the Dutch GP-setting. These models potentially mitigate the aforementioned shortcomings of MetS, because they quantify the absolute CVD-risk, and include additional relevant cardiovascular determinants, such as age, sex and smoking status. Unfortunately, these models are not adapted to the increased CVD-risk psychiatric patients experience.

Framingham predicts the 10-year morbidity risk to develop the first cardiovascular event, expressed as a percentage. It has a median AUC of 77% (95% CI: 58%–84%), based on 28 studies [28]. Rigal and colleagues demonstrated in their meta-analysis that individuals with a severe mental illness (SMI) had Framingham coronary scores that were 1.6 fold higher than in control subjects from the general population [29], confirming the elevated risk of these patients. However, there are indications that Framingham overestimates the risk of coronary heart disease when the formula is applied to European populations [30]. In addition, not all studies agree that Framingham is superior to MetS with regard to CVD-risk prediction [31–33].

The second continuous scoring model, SCORE, predicts the 10-year CVD-mortality risk expressed as a percentage [27]. The median AUC of SCORE for CVD-prediction was 75% (95% CI: 62%–91%), based on 28 studies [28]. Its implementation is recommended by the Dutch College of General Practitioners in their cardiovascular risk management (CVRM) guideline, as well as by the European Association for Cardiovascular Prevention & Rehabilitation [34]. *Absolute* SCORE-risks have been studied in the SMI-population, yielding mean risks between 0.9% and 1.8% [35]. On the other hand, the European Psychiatric Association (EPA) recommends in their Position Statement the use of *relative* SCORE-risks, especially in the younger SMI-population. According to the smoking habits, systolic blood pressure, and total cholesterol of patients with SMI, the risk of fatal cardiovascular disease is compared to the general population (that is, people without SMI of the same age with normal risk factors) [36]. However, the Statement does not mention cut-off values for which relative risks prophylactic treatment should be initiated. In addition, some studies argue that SCORE underestimates the true CVD-risk [37, 38].

The large-scale studies performed to establish 10-year CVD-mortality and morbidity-risks for SCORE and Framingham, were carried out in the general population, which may limit the generalizability of the calculated risks to the psychiatric population. Risk-calculators calibrated or adjusted to the SMI-population can potentially improve the risk-estimation and may better address underestimations [39, 40]. The PRIMROSE desk and lipid-models [41] are among the few models that take the CVD risk-increasing effects of SMI-diagnosis, psychosocial and -pharmacological treatment into account [2, 11] by adding these risk factors to the algorithm, besides the traditional, well-established cardiovascular predictors [41, 42]. The PRIMROSE models were validated in the UK in a SMI-population aged 30 to 90 years, and estimate the 10-year risk percentage to incur the first cardiovascular event. The authors described AUCs around 80% (range 95% CI: 74%–83%) for CVD prediction [41]. Perry et al., however, demonstrated that PRIMROSE systematically underpredicted the CVR in adolescents with, or at risk for, psychosis [43].

To summarize, there are at least four different CVD-risk assessment-models in use by clinicians for psychiatric patients with a range of psychiatric problems, each with advantages and drawbacks. Currently, it is unclear (1) whether the use of any of the four CVR-assessment models under study, when applied to the same patient, leads to a similar CVD-risk and subsequent management, and (2) whether there is any evidence suggesting the superiority of one CVD-risk assessment method over another with respect to sensitivity and specificity in predicting CVD events, notably for psychiatric patients in secondary mental healthcare [44]. In light of the theoretical situation that the use of a suboptimal CVR-evaluator may contribute to an increase in excess life years lost due to heart diseases in mental health patients because of underestimated risk and as a consequence undertreatment [19, 45], this is not a trivial question. It is currently unclear whether the assessment methods used in general healthcare (mostly Framingham, SCORE), when used in mental health care, are equally predictive as PRIMROSE which has been specifically designed for psychiatric populations. Moreover, these models need comparison with the criteria of presence/absence of MetS, which to date seems to be the most commonly used CVR-estimator in mental healthcare. Hence, the aims of this study are to cross-sectionally compare current CVR-monitoring systems in a large and heterogenous group of mental health care outpatients. We aimed to investigate whether there is a *systematic difference* in the agreement between the outcomes of MetS, Framingham, SCORE, modified SCORE, PRIMROSE lipid, and PRIMROSE desk, within the same person. Second, we aimed to determine the *strength* of the agreements between these assessment methods. Finally, we evaluated the strength of the correlations between the total number of MetS criteria that are met, and the various CVD-risk predictions.

## Methods

### Study design and population

We used two databases: PHAMOUS (‘PHArmacotherapy Monitoring and Outcome Survey’) and MOPHAR (‘Monitoring Outcomes of psychiatric PHARmacotherapy’) to answer our research questions. Both databases were used separately, instead of a combined one, in order to verify the transdiagnostic consistency and generalizability of the findings. The PHAMOUS and MOPHAR study protocols have been described in detail elsewhere [46, 47]. In short, PHAMOUS (*N* = 13,215 patients with a combined total of 42,776 measurements as of November 2021) has been including patients aged 18 years and older since 2006, originally focusing on schizophrenia, schizoaffective disorder, or other psychotic disorders, at four mental health facilities in the Northern Netherlands. For the last couple of years, PHAMOUS has been including SMI diagnosed by DSM-criteria in a broader diagnostic spectrum. MOPHAR (*N* = 2098 patients with a combined total of 2,986 measurements) started a decade later, and includes adults with any psychiatric diagnosis from outpatient departments of mental health facilities in the Northern Netherlands.

Patients were eligible for our study if they were aged between 40–70 years, because (i) all of our CVD risk models have been validated for this age range; (ii) CVR is substantially age-related, it is generally acknowledged that CVR-monitoring is most useful from age 40 on in the general population. Patients were included if they completed at least one of the yearly somatic assessments. Exclusion criteria were (1) a history of CVD, because we selected CVD-risk models that target primary prevention, (2) the presence of diabetes mellitus, because diabetic patients are automatically classified as patients with a high to very high CVD risk, and should be assessed with other risk engines, such as ADVANCE [48].

Patients are screened yearly by the MOPHAR or PHAMOUS-program, on somatic, and psychiatric (co)morbidities, disease severity, psychosocial functioning, current medication usage, registration of side effects. Further, a basic physical examination (e.g. measurement of waist circumference and blood pressure) is carried out by trained nurses, and laboratory measurements are collected, including blood lipids (i.e. low and high-density lipoprotein (LDL and HDL), total cholesterol, triglycerides) and fasting glucose. PHAMOUS and MOPHAR are highly similar with respect to the somatic parameters assessed, and differ on assessment of symptom parameters, as a result of differences between their populations (MOPHAR targeting mostly patients with affective and anxiety disorders, PHAMOUS mostly psychotic patients). If the same patient had multiple risk score and MetS assessments, we included every complete set of data points. We used statistical tests and measurements that reliably account for the paired nature of the risk comparisons [49]. Using multiple data points has the advantage that it accommodates the detection of intraindividual changes between assessments (regarding treatment, or risk factor exposition, which could induce alterations in MetS-status and/or CVD-risks).

### Measurements

**Fig. 1:**
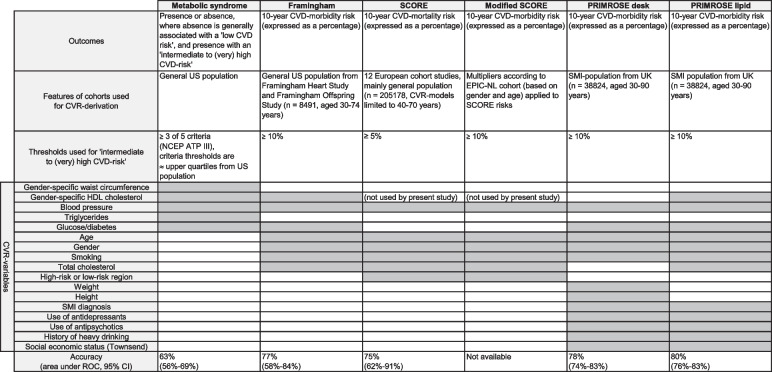
Summary of the outcomes, features of the cohorts used for CVR-derivation, applied thresholds, CVR-variables, and accuracy of the various CVR-models (i.e., MetS, Framingham, SCORE, modified SCORE, PRIMROSE desk, and PRIMROSE lipid)

A detailed overview of the various models, and addressed CVD-predictors, can be found in Fig. 1 and Appendix 1.

#### Metabolic syndrome

Patients are considered to have MetS if they fulfil three or more of the following criteria [50]: 1) waist circumference ≥ 88/102 cm (female/male); 2) systolic blood pressure ≥ 130 mmHg or diastolic blood pressure ≥ 85 mmHg or receiving antihypertensive drug treatment; 3) HDL-cholesterol < 1.30/1.03 mmol/L (female/male) or receiving lipid-lowering drugs; 4) triglycerides ≥ 1.7 mmol/L or receiving lipid-lowering drugs; and 5) fasting glucose ≥ 6.1 mmol/L or receiving antidiabetic medication(s). There are 16 unique combinations of MetS, to which we collectively refer as ‘MetS-profiles’.

#### Framingham

The Framingham risk-score estimates the 10-year CVD-morbidity risk, and is derived from large scaled population-based North-American samples without CVD, aged 30–74 years. Because our total cholesterol and HDL-cholesterol results were expressed in SI-units (i.e. mmol/L), we multiplied our laboratory results with 38.6698 to convert these to mg/dL [51] to be entered in the risk model. Patients with a Framingham risk-score of at least 10% were considered to have a substantially increased cardiovascular risk [52].

#### SCORE

The SCORE-formula is derived from individuals of the general European population aged 40–70 years, without known CVD. Patients with a SCORE between 5%-10% are considered to have a moderate to high risk, ≥ 10% a very high risk [53]. To compare the various morbidity risks, we applied multipliers specified by the Dutch CVRM-guideline to convert the SCORE-mortality risk-score to estimated morbidity-scores (‘modified SCORE’). ‘Modified SCORE’ risks of at least 10% were considered to be substantial.

#### PRIMROSE

The PRIMROSE desk and lipid-models entail 10-year CVD-morbidity risk-estimations from a study conducted in the UK in a SMI-population aged 30 to 90 years. Because we did not have sufficient information on social deprivation, we instead averaged the ‘best’ and ‘worst case risk scenario’ by assuming the lowest, respectively, highest Townsend quintiles in the PRIMROSE-formulas. A cut off value of ≥ 10% was used for the PRIMROSE-risk to be considered substantial.

### Statistical analyses

We used R version 4.1.2 [54] to determine the CVR-assessments by using ‘available case’-analyses. A description of the prediction-formulas can be found in Appendix 1.

#### Comparisons between MetS-status and dichotomized CVD-risk categories

First, we compared the agreement between these methods as present versus absent. Because the binary MetS is a nominal variable (i.e. the absence of MetS is generally associated with a ‘low CVD risk’, the presence of MetS with an ‘intermediate to (very) high CVD risk’), we dichotomized the continuous outcomes of Framingham, SCORE, modified SCORE, and PRIMROSE models, to the same CVD-risk outcome categories as MetS. ‘Modified SCORE’, Framingham, and PRIMROSE risks of ≥ 10% were considered to be an ‘intermediate to (very) high CVD risk’. For SCORE we used a cut-off of 5%, in line with the Dutch CVRM guideline.

We determined whether there was a *systematic difference* between the agreement of the presence or absence of MetS and each of the dichotomized risk-categories, by performing McNemar’s tests with the mcnemar.test-function of R’s *stats*-package [49, 55]. Subsequently, we assessed the *strength* of agreement between these comparisons by measuring Cohen’s kappa *κ* with 95% confidence intervals by using the Kappa.test-function of R’s *fmsb*-library [49]. Kappa can be interpreted as follows: below 0 is poor agreement, 0–0.20 slight, 0.21–0.40 fair, 0.41–0.60 moderate, 0.61–0.80 substantial, and > 0.80 almost perfect agreement [56].

#### Mean CVD-risks stratified by number of MetS-criteria met, and their correlation

Furthermore, we stratified the mean CVD-risks by the number of MetS-criteria that were met, and by ‘MetS-profile’. The correlation between the number of MetS-criteria and absolute risk-scores, was calculated by Kendall’s tau with 95% confidence intervals (by using R’s KendallTauB-function in the *DescTools*-package). We used Botsch’s guidelines for the interpretation of the magnitude of *τ*, that is: |*τ*|< 0.10 = very weak, |*τ*| = 0.10–0.19 = weak, |*τ*| = 0.20–0.29 = moderate, and |*τ*|≥ 0.30 = strong strength of association between the number of MetS-criteria and CVD-risk score [57].

#### Pairwise comparisons between continuous CVD-risks

The assessment of the degree of agreement between the continuous risk-outcomes of Framingham, modified SCORE, and PRIMROSE lipid and desk models, took place in a pairwise fashion and was quantified by Lin’s concordance correlation-coefficients *ρ* (by the CCC-function in R’s *DescTools*-library). McBride’s cut-off values were used to assess the strength-of-agreement [58], i.e. almost perfect agreement if *ρ* > 0.99, substantial agreement if *ρ* is 0.95–0.99, moderate if *ρ* is 0.90–0.95, and poor strength of agreement if *ρ* < 0.90.

## Results

**Fig. 2:**
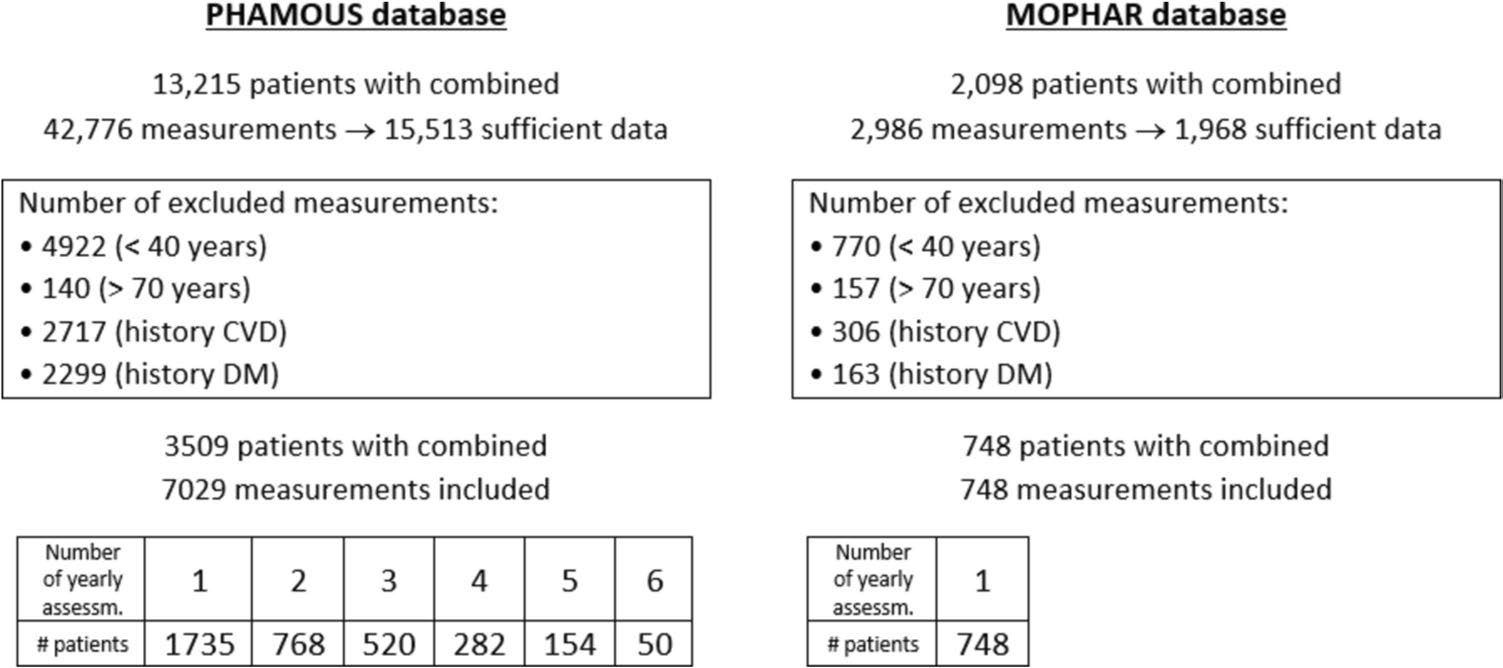
Flowcharts with reasons for exclusion, total amount of included measurements (as well as number of unique patients), and distribution of frequency of yearly assessments, from the PHAMOUS-, and MOPHAR-database

Of the 42,776 combined measurements of the PHAMOUS-database, 36% had sufficient information to be eligible for inclusion; which was 66% for the MOPHAR-database. Most of the measurements were excluded based on age (< 40; > 70 years), followed by presence of a history of CVD, and/or DM. Almost half of the eligible PHAMOUS-measurements, from 3509 unique patients, met our inclusion criteria, compared to more than one third of MOPHAR’s (see Fig. 2).

For a more detailed comparison between the in- and excluded groups, as well as missing values, see Supplementary material 1. Characteristics of the included samples can be found in Table 1.

**Table 1:** Descriptive table with characteristics of measurements satisfying our inclusion criteria (i.e. from patients without CVD or diabetes, and aged between 40 and 70 years) in the PHAMOUS (*n* = 7029 measurements from 3509 unique patients), and MOPHAR-database (*n* = 748 measurements from 748 unique patients)

	**PHAMOUS**	**MOPHAR**
	***n* = 7029** **measurements**	***n* = 748** **measurements**
Sex		
Male (n (%))	4061 (57.8%)	309 (41.3%)
Female (n (%))	2968 (42.2%)	439 (58.7%)
Age (in years, mean ± sd)	51.4 ± 7.27	52.2 ± 7.95
Waist circumference (in cm, mean ± sd)	102 ± 15.0	100 ± 13.8
Systolic blood pressure (in mmHg, mean ± sd)	129 ± 18.1	133 ± 18.1
Diastolic blood pressure (in mmHg, mean ± sd)	83.9 ± 11.3	84.0 ± 11.0
High density lipoproteine (HDL, in mmol/L, mean ± sd)	1.34 ± 0.51	1.44 ± 0.46
Total cholesterol (in mmol/L, mean ± sd)	5.18 ± 1.12	5.35 ± 1.03
Triglycerides (in mmol/L, mean ± sd)	1.76 ± 1.08	1.52 ± 0.80
Glucose (in mmol/L, mean ± sd)	5.69 ± 1.09	5.55 ± 0.92
Metabolic Syndrome n (%)	2084/6899 (30.2%)	160/748 (21.4%)
Nr. Of Metabolic Syndrome criteria		
0 (n (%))	1086 (15.7%)	90 (12.0%)
1 (n (%))	1857 (26.9%)	250 (33.4%)
2 (n (%))	1872 (27.1%)	248 (33.2%)
3 (n (%))	1062 (15.4%)	95 (12.7%)
4 (n (%))	792 (11.5%)	51 (6.82%)
5 (n (%))	230 (3.33%)	14 (1.87%)
Current smoker (n (%))	3963 (56.4%)	217 (35.2%)
Ex smoker (n (%))	1333 (19.1%)	179 (29.1%)
Excessive alcohol use (n (%))	2788 (40.0%)	25 (6.00%)
Weight (in kg, mean ± sd)	87.3 ± 19.3	83.0 ± 17.9
Height (in cm, mean ± sd)	177 ± 9.76	174 ± 10.2
Bipolar diagnosis (n (%))	611 (9.54%)	141 (20.1%)
Other psychotic disorder (n (%))	4025 (62.8%)	34 (4.84%)
Unspecified Severe Mental Illness (n (%))	1769 (27.6%)	527 (75.1%)
Use of antidepressive medication (n (%))	2410 (35.4%)	338 (45.2%)
Second generation antipsychotics (n (%))	3696 (52.6%)	208 (27.8%)
First generation antipsychotics (n (%))	1426 (20.3%)	16 (2.14%)
Framingham risk in % (mean ± sd): 10 year CVD morbidity risk	12.5% ± 9.69%	10.6% ± 8.54%
SCORE risk in % (mean ± sd): 10 year CVD mortality risk	1.76% ± 2.25%	1.38% ± 1.85%
Modified SCORE risk in % (mean ± sd): 10 year CVD morbidity risk	8.65% ± 9.37%	7.14% ± 8.04%
PRIMROSE desk risk in % (mean ± sd): 10 year CVD morbidity risk	4.27% ± 2.97%	2.81% ± 2.17%
PRIMROSE lipid risk in % (mean ± sd): 10 year CVD morbidity risk	3.84% ± 2.90%	2.52% ± 2.18%

Almost one third of the PHAMOUS-measurements met the MetS-criteria (30.2%; 95% CI: 29.1%–31.3%), compared to more than one in five in MOPHAR (21.4%; 95% CI: 18.5%–24.5%). The majority of the measurements that fulfilled MetS, satisfied three criteria (1062/2084 = 51% for PHAMOUS, vs. 95/160 = 59% for MOPHAR, see Table 1).

Cross-tables with frequencies and percentages of (dis)agreement between the outcomes of the CVR-assessment methods (based on binary risk classifications from six methods) are depicted in Table 2. The largest range between these percentages occurred in the measurements fulfilling MetS-criteria, both in the PHAMOUS (i.e. range of percentages of (dis)agreement between CVR-assessment methods with MetS belonging to the *highest* CVD-risk category was 7.2%-62.1%, vs. *lowest* CVD-risk-category: 37.9%-92.8%), and in the MOPHAR-database (i.e. range highest CVD-risk category: 3.7%-60.4%, vs. lowest CVD-risk-category: 39.6%-96.3%).

**Table 2:**
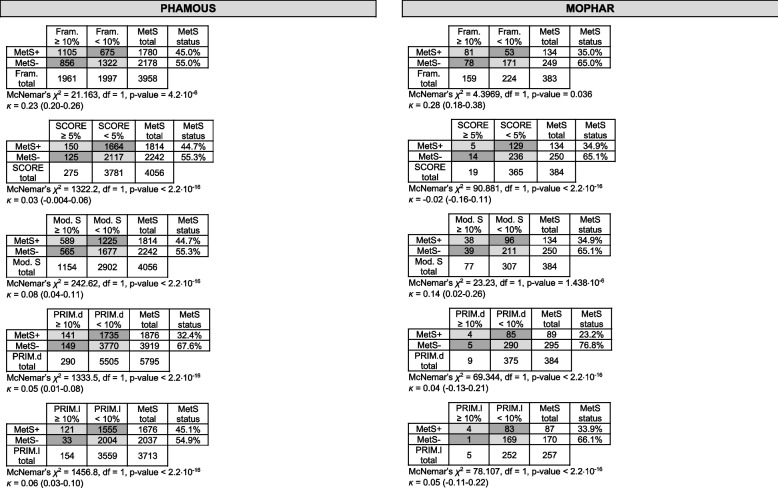
Cross tables with concordant (light grey), and discordant (dark grey) frequencies of binary CVD-risk classifications from six assessment methods (i.e., dichotomous MetS-status (= presence vs. absence of MetS) vs. CVD-risk categories (= ‘intermediate-(very) high risk’ vs. ‘low risk’) based on thresholds of continuous Framingham, SCORE, Modified SCORE, PRIMROSE desk, and PRIMROSE lipid scores)

*Abbreviations*: *MetS+* presence of Metabolic Syndrome, *MetS-* absence of Metabolic Syndrome, *Fram.* Framingham risk score, *SCORE* SCORE risk score, *Mod. S* Modified SCORE risk score, *PRIM. D* PRIMROSE desk risk score, *PRIM. L* PRIMROSE lipid risk score, *MetS st.* Metabolic Syndrome status, *df *degrees of freedom

### MetS-status versus dichotomized CVD-risk categories in PHAMOUS and MOPHAR

We evaluated whether there was a *systematic difference* between MetS-status and the dichotomized CVD-risk categories in the PHAMOUS-, and MOPHAR-database separately. Indeed, the McNemar’s chi-squared *p*-values indicated significant differences between the binary risk-classifications of the CVR-assessment methods (all *p* < 0.036; Table 2).

The *extent of agreement* was ‘fair’ between the categorical risk-classifications by MetS and Framingham (range *κ* = 0.23–0.28 (95% CI = 0.18–0.38)), all the other comparisons agreed ‘poorly’ to ‘slightly’ (*κ*’s ≤ 0.14, range 95% CI: -0.16–0.26), as shown in Table 2.

### Mean CVD-risks by number of MetS-criteria, and their correlation

Next, we examined the mean CVD-risks by the number of MetS-criteria that were fulfilled, as well as their correlations.

#### PHAMOUS

**Fig. 3:**
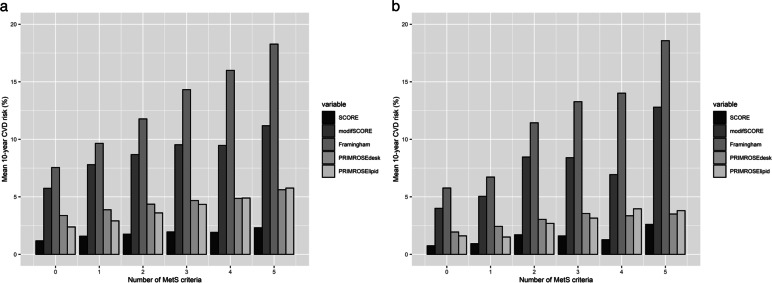
Mean 10-year CVD-risks stratified by the number of MetS criteria, and risk-model **a** PHAMOUS, **b **MOPHAR

The lowest mean 10-year CVD-morbidity risks of the PHAMOUS-database occurred when none of the MetS-criteria were fulfilled, ranging from 2.38% (95% CI: 2.21%–2.55%; PRIMROSE lipid), to 7.55% (95% CI: 7.00%–8.10%; Framingham; see Fig. 3A).

The highest mean risks occurred when all of the MetS-criteria were fulfilled, with a minimum of 5.61% (95% CI: 5.10%–6.14%; PRIMROSE desk), and a maximum of 18.28% (95% CI: 16.72%–19.83%; Framingham). We refer to Supplementary material 2 for subgroup CVD-risk analyses per number of MetS-criteria fulfilled and MetS-profile.

There was a moderate positive correlation between the number of MetS-criteria and Framingham risk-scores (Kendall’s *τ* = 0.25, 95% CI: 0.23–0.27), as well as between the number of MetS-criteria and PRIMROSE lipid-scores (*τ* = 0.26, 95% CI: 0.24–0.28). The other correlations were all (very) weak, with *τ*’s < 0.13.

#### MOPHAR

The range of the lowest mean 10-year CVD-morbidity risks of the MOPHAR-database, was 1.51% (for the PRIMROSE lipid-group; 95% CI: 1.24%-1.78%) to 5.77% in the Framingham-group that had zero MetS-criteria (95% CI: 4.53%–7.00%).

The highest mean risks occurred when all of the MetS-criteria were fulfilled, with a maximum of 18.57% in the Framingham-group (95% CI: 9.49%–27.65%; see Fig. 3B).

There was a strong positive correlation between the number of MetS-criteria and Framingham risk-scores (τ = 0.34, 95% CI: 0.28–0.40), as well as between the number of MetS-criteria and PRIMROSE lipid-scores (τ = 0.32, 95% CI: 0.24–0.40). Other correlations were weak with τ’s < 0.19.

### Pairwise comparisons between continuous CVD-risk outcomes in the PHAMOUS and MOPHAR databases

We found a moderate strength-of-agreement between the continuous risk outcomes of the PRIMROSE lipid and desk-model in the MOPHAR-database (*ρ* = 0.91 (95% CI: 0.89–0.93), Fig. 3B in Suppl. 1). The pairwise comparisons of continuous CVD-risk outcomes of PHAMOUS, and the other pairwise comparisons of MOPHAR, all agreed poorly with *ρ*’s between 0.20 and 0.85.

## Discussion

This study directly compared cardiovascular risk-assessment methods as used in psychiatric practice (MetS, PRIMROSE-models), as well as in GP-practices (SCORE, and Framingham-model), across two large transdiagnostic and heterogeneous psychiatric patient samples receiving secondary care. CVD-risk was on average 2.52%-4.27% for psychiatric practice methods, and 1.38%-12.5% for GP-practice methods in these two samples. We found evidence for systematic disagreement between the categorical methods of CVD-risk assessment. Furthermore, the strength-of-agreement between these binary CVR-assessment methods was generally poor to slight. Only MetS and Framingham corresponded slightly, as evidenced by fair agreement in both study groups.

Our findings provide supporting evidence that CVD-risk is elevated in psychiatric populations. Indeed, the mean Framingham and SCORE-risks in our samples were comparable to the estimated risks in studies performed in bipolar [59–61], depressive [62], and schizophrenic populations [63, 64]. Additionally, we found support for higher CVD-risks in more severely ill patients, as demonstrated by higher mean risks in the PHAMOUS-patients compared to the MOPHAR-group. This can be explained by a higher prevalence of SMI in the PHAMOUS-cohort, their longer illness duration, less favourable lower mean HDL, higher triglycerides, higher smoking and excessive drinking frequencies, and higher use of antipsychotics. Accurate assessment of CVD-risk is therefore of high importance in this group.

However, we found that CVD-risk assessments lead to uneven risk predictions, especially for more severely ill patients. The predominantly poor degree of agreement between the continuous CVR-comparisons is in accordance with the findings of Berry and colleagues, who reported similar *ρ*’s between 0.54 and 0.77 for Framingham, PRIMROSE lipid, and PRIMROSE desk [44]. Indeed, the largest individual disparity between continuous CVR-outcomes occurred in the PHAMOUS-database, where the ‘modified SCORE’ predicted a CVD-risk of 100%, versus only 5.94% by the PRIMROSE lipid-model, for the same 63-year old smoking male with an unspecified SMI and MetS (Fig. 4B, Suppl. 1). This can be explained by the greater emphasis by the PRIMROSE lipid-model on the CVD-protective effect of his high HDL (i.e. 2.5 mmol/L), whereas the modified SCORE-model more strongly emphasizes his high systolic blood pressure (i.e. 185 mmHg), and age (i.e. 63 years old). This indicates that various CVD-risk assessment methods, used in different clinical settings, yield uneven risk predictions.

Only in a few instances, we found some agreement between CVD-risk assessment methods, but only between those that are used in the same clinical setting (i.e., in psychiatric practice). The PRIMROSE lipid and PRIMROSE desk-model had at most a moderate strength-of-agreement when continuous CVD-outcomes were compared, in both samples. Moderate strength-of-agreement can be expected between the PRIMROSE-models, because they share most parameters, and have been derived from, and validated in, the same SMI-sample, and should therefore produce comparable risk-predictions. Secondly, moderate to strong correlations were found between the number of MetS-criteria and Framingham risk-scores, as well as between the number of MetS-criteria and PRIMROSE lipid-scores. These results are in line with previous findings of Wilson et al., and Wannamethee and colleagues, who demonstrated positive associations between the number of MetS-criteria fulfilled and increased CVD-risks measured by Framingham [33, 65].

Considering the inconsistencies in reported CVR-criteria and subsequent risk-estimations between the various models, we can conclude that persons with a psychiatric disorder have a substantial chance to attain a varying risk profile depending on the context (general practice versus psychiatric care) and related CVR-risk management-model in which they are monitored and treated. As a consequence, psychiatric patients might not receive necessary treatments due to significant underpredictions of the CVD-risk (e.g., by SCORE or PRIMROSE), or receive unnecessary treatments due to overpredicted CVD-risks (e.g., by Framingham). The differences in assessed risks highlight the necessity to provide recommendations on the preferred model. Such recommendations depend on certain quality criteria.

Most importantly, a CVR-assessment model should include relevant predictors to optimize sensitivity and specificity to reliably predict CVD-morbidity. With this cross-sectional study, we demonstrated that various CVR-assessment models yield unequal risk predictions; the next step should involve the investigation of the relative contribution of each of the predictors in each model, and the comparison of the individual predicted risks with the actual occurrence of CVD-events within 10 years. Besides the ‘traditional’ cardiovascular risk factors such as demographic, inflammatory, metabolic, physiologic, and lifestyle factors [66], one should also include relevant environmental (including psychosocial and psychological) factors in the model that are known to increase the cardiovascular risk, especially in the psychiatric population (such as insomnia, vital exhaustion, depression, anxiety, anger/hostility, social isolation/loneliness, optimism, psychological distress, adverse childhood experiences; see Supplementary Table 2 of ref. [40]). Future research should therefore focus on combining longitudinal risk assessments (over ≥ 10 years) with the occurrence of CVD-events in large psychiatric populations, to enable well-founded recommendations on which (recalibrated) CVD-risk model should be employed in the psychiatric population.

In the meantime, as described by Perry et al., we advise clinicians to use MetS because it is not restricted to older individuals (with SCORE: ≥ 40 years), and is therefore more suitable for cardiometabolic risk assessment in the younger psychiatric population [43], whereas SCORE [67] and PRIMROSE [43] underpredict. Reversely, Framingham overpredicts this CV risk (as shown by Danish, German, and Dutch studies [68–70]). The underprediction is mainly caused by age being more strongly weighted than other risk factors; ‘older’ CVR-estimators (such as Framingham) overpredict the risk due to a decrease in coronary heart disease incidence and mortality rates in the last four decades [30]. However, earlier initiation of somatic treatment nowadays (e.g., with statins, and anti-hypertensive medication) could be partly responsible for this overprediction as well [71]. Furthermore, MetS has the advantage that it is relatively easy and fast to use, and it does not require rather complicated prediction formulas which can be cumbersome in the daily clinical practice.

### Strengths and limitations

Strengths of our study include the relatively large naturalistic samples of psychiatric patients, which included patients with diverse diagnoses and severity levels. Furthermore, the use of two databases allowed us to substantiate the generalizability of the results across populations.

Limitations include the applied age-restriction, excluding patients that are younger than 40 (or older than 70). The younger population is not routinely screened for CVD, even though psychiatric disorders most often originate in early adulthood, and presence of a psychiatric disorder should be considered as an independent cardiovascular risk factor, also at earlier age [13, 43, 72–75].

Second, we employed ‘available case’-analyses even though not all missing data were expected to be missing completely at random, because the missingness is probably influenced by some patient characteristics, such as disease severity. This is, however, largely compensated by the considerable sample size [76].

Third, we cannot exclude some biases. Patients who are experiencing a severe exacerbation of their psychiatric illness are, for instance, less likely to be assessed, which can induce response-bias. Detection-bias cannot be ruled out either, because practitioners may encourage medicated, or more severely ill patients, to participate in the monitoring programs more strongly than unmedicated, or less severely ill patients.

## Conclusions

To conclude, the commonly used CVD-risk assessments generally show strong disagreement. Given that GPs and psychiatrists rely on different methods, this means that CVD-assessments lead to disparate results. As a consequence, psychiatric patients might not receive necessary treatments due to significant underpredictions of the CVD-risk (e.g., by SCORE or PRIMROSE), or receive unnecessary treatments due to overpredicted CVD-risks (e.g., by Framingham). Future research should compare the quality of the predictive performances of the established CVD-risk models, preferably calibrated to psychiatric patients aged 18 and above. We favour the implementation of a single CVD-risk model for psychiatric patients by the healthcare practitioners, on the condition that the model performs equally well in the various settings. In this way we can, hopefully, start to close the cardiovascular health gap between the psychiatric and non-psychiatric population.

## Supplementary Information

**Additional file 1:** **Supplementary document 1.** [Quadackers_suppl1.docx; contains descriptive tables by ‘inclusion’ and missing status, pairwise comparisons of continuous outcomes of CVD-risk models, and appendix 1 (specifies which MetS criteria and riskfunctions we employed].

**Additional file 2:** **Supplementary document 2.** [Quadackers_suppl2.docx; contains the mean CVD-risks stratified per MetS-criterium and MetS-profile].

## Data Availability

The datasets used and/or analysed during the current study are available from the corresponding author on reasonable request.

## Abbreviations

CVD: Cardiovascular diseases
CVR: Cardiovascular risk
MetS: Metabolic syndrome
GP: General practitioner
PRIMROSE: PRedIction and Management of cardiovascular Risk in peOple with SEvere mental illnesses
PHAMOUS: Pharmacotherapy Monitoring and Outcome Survey
MOPHAR: Monitoring Outcomes of Psychiatric Pharmacotherapy
SCORE: Systematic Coronary Risk Evaluation
CVRM: Cardiovascular risk management
AUC: Area under the receiver-operating characteristic curve
NHG: 'Nederlands Huisartsen Genootschap' (The Dutch College of General Practitioners)
SMI: Severe mental illness
DSM: Diagnostic and Statistical Manual of Mental Disorders
LDL: Low-density lipoprotein
HDL: High-density lipoprotein
