# Supplementary material for: Cardiovascular risk assessment methods yield unequal risk predictions: a large cross-sectional study in psychiatric secondary care outpatients
Source: BMC Psychiatry. 2023 Jul 24;23:536. doi: 10.1186/s12888-023-05022-1 (PMC10367364; doi:10.1186/s12888-023-05022-1)
Supplement: Supplementary file 1 — Additional file 1: Supplementary document 1. [Quadackers_suppl1.docx; contains descriptive tables by ‘inclusion’ and missing status, pairwise comparisons of continuous outcomes of CVD-risk models, and appendix 1 (specifies which MetS criteria and riskfunctions we employed]. [file 12888_2023_5022_MOESM1_ESM.docx]

**Contents supplementary material 1**

I. Descriptive tables by ‘inclusion’ and missing status

1. PHAMOUS 2

2. MOPHAR 11

II. Pairwise comparisons of continuous outcomes of CVD-risk models 20

Appendix 1

1. Metabolic syndrome diagnosis (based on ATP III criteria from NCEP) 23

2. Framingham risk score formula 23

3. SCORE risk score formula 24

4. PRIMROSE risk score formula 25

**I. Descriptive tables by ‘inclusion’ and missing status**

1.PHAMOUS

--------Summary descriptives table by 'inclusion'---------

(0: excl.) (1: included)

0 1 p.overall

N=8484 N=7029 **Abbreviations**

¯¯¯¯¯¯¯¯¯¯¯¯¯¯¯¯¯¯¯¯¯¯¯¯¯¯¯¯¯¯¯¯¯¯¯¯¯¯¯¯¯¯¯¯¯¯¯¯¯ _____________

gender: <0.001

0 5267 (62.1%) 4061 (57.8%) 0 = male

1 3217 (37.9%) 2968 (42.2%) 1 = female

wc 103 (17.2) 102 (15.0) <0.001 wc = waist circumference

sysRR 130 (18.1) 129 (18.1) 0.477 sysRR = systolic blood pres.

diaRR 82.5 (11.5) 83.9 (11.3) <0.001 diaRR = diastolic blood pr.

antihyp: <0.001 antihyp = antihypertensive

0 6105 (74.8%) 6238 (91.5%) 0 = no treatment

1 2060 (25.2%) 577 (8.47%) 1 = treatment

HDL 1.23 (0.48) 1.34 (0.51) <0.001 HDL = high density lipoprot.

statine: <0.001 (in mmol/L)

0 6332 (77.6%) 6101 (89.5%) 0 = no statine treat.

1 1833 (22.4%) 714 (10.5%) 1 = statine treat.

triglyc 1.90 (1.23) 1.76 (1.08) <0.001 triglyc = trigylcerides
 (in mmol/L)

fibr: <0.001 fibr = fibrate use

0 6332 (77.6%) 6101 (89.5%) 0 = no treatment

1 1833 (22.4%) 714 (10.5%) 1 = treatment

gluc 6.40 (2.37) 5.69 (1.09) <0.001 gluc = glucose (mmol/L)

diabmed: <0.001 diabmed = antidiabetic

0 6444 (78.9%) 6761 (99.2%) 0 = no treatment

1 1721 (21.1%) 54 (0.79%) 1 = treatment

MetS.crit: <0.001 MetS.crit = number of

0 1417 (17.1%) 1086 (15.7%) criteria Metabolic Syndrome

1 1792 (21.6%) 1857 (26.9%)

2 1636 (19.8%) 1872 (27.1%)

3 1102 (13.3%) 1062 (15.4%)

4 1157 (14.0%) 792 (11.5%)

5 1174 (14.2%) 230 (3.33%)

MetS.prof: <0.001 MetS.prof = Metabolic

BGH 17 (0.50%) 16 (0.77%) Syndrome profile, where

BGHT 216 (6.29%) 46 (2.21%) B = high blood pressure

BGHTW 1174 (34.2%) 230 (11.0%) G = high glucose

BGHW 67 (1.95%) 62 (2.98%) H = low HDL

BGT 29 (0.84%) 37 (1.78%) T = high triglycerides

BGTW 99 (2.88%) 99 (4.75%) W = high waist circumference

BGW 296 (8.62%) 137 (6.57%)

BHT 207 (6.03%) 175 (8.40%)

BHTW 559 (16.3%) 506 (24.3%)

BHW 111 (3.23%) 135 (6.48%)

BTW 124 (3.61%) 210 (10.1%)

GHT 102 (2.97%) 50 (2.40%)

GHTW 216 (6.29%) 79 (3.79%)

GHW 31 (0.90%) 28 (1.34%)

GTW 37 (1.08%) 16 (0.77%)

HTW 148 (4.31%) 258 (12.4%)

age 41.5 (13.6) 51.4 (7.27) 0.000

totalchol 4.73 (1.13) 5.18 (1.12) <0.001 total cholesterol (mmol/L)

cursmoker: 0.017 current smoker

0 3538 (41.7%) 3066 (43.6%) 0 = no

1 4946 (58.3%) 3963 (56.4%) 1 = yes

DM: 0.000 Diabetes Mellitus present

0 6185 (72.9%) 7029 (100%) 0 = no

1 2299 (27.1%) 0 (0.00%) 1 = yes

histCVD: 0.000 History of cardiovascular

0 5767 (68.0%) 7029 (100%) 0 = no diseases

1 2717 (32.0%) 0 (0.00%) 1 = yes

Framingham 12.7 (14.5) 12.5 (9.69) 0.494 Framingham risk score (%)

SCORE 1.32 (2.82) 1.76 (2.25) <0.001 SCORE risk score (%)

modifSCORE 11.4 (11.8) 8.65 (9.37) <0.001 modified SCORE risk (%)

weight 90.6 (20.9) 87.3 (19.3) <0.001

height 177 (10.3) 177 (9.76) 0.023

exsmoker: 0.004 Ex smoker

0 6849 (82.7%) 5661 (80.9%) 0 = no

1 1429 (17.3%) 1333 (19.1%) 1 = yes

antidep: 0.017 Use of antidepressants

0 5430 (66.5%) 4405 (64.6%) 0 = no

1 2735 (33.5%) 2410 (35.4%) 1 = yes

alcohol: 0.459 Excessive alcohol use

0 4985 (59.4%) 4185 (60.0%) 0 = no

1 3405 (40.6%) 2788 (40.0%) 1 = yes

bipol: <0.001 Diagnosis of bipolar disord.

0 6844 (92.7%) 5794 (90.5%) 0 = no

1 540 (7.31%) 611 (9.54%) 1 = yes

othpsychosis: 0.015 Diag. other psychotic dis.

0 2595 (35.1%) 2380 (37.2%) 0 = no

1 4789 (64.9%) 4025 (62.8%) 1 = yes

unspecSMI: 0.797 Unspecified severe mental

0 5329 (72.2%) 4636 (72.4%) 0 = no illness

1 2055 (27.8%) 1769 (27.6%) 1 = yes

secgenAP: 0.193 Second generation anti-

0 3933 (46.4%) 3333 (47.4%) 0 = no psychotics

1 4551 (53.6%) 3696 (52.6%) 1 = yes

firstgenAP: 0.001 First generation anti-

0 6943 (81.8%) 5603 (79.7%) 0 = no psychotics

1 1541 (18.2%) 1426 (20.3%) 1 = yes

PRIMROSEdesk 4.72 (6.41) 4.27 (2.97) <0.001 PRIMROSE desk risk score (%)

PRIMROSElipid 4.24 (5.60) 3.84 (2.90) <0.001 PRIMROSE lipid risk score(%)

¯¯¯¯¯¯¯¯¯¯¯¯¯¯¯¯¯¯¯¯¯¯¯¯¯¯¯¯¯¯¯¯¯¯¯¯¯¯¯¯¯¯¯¯¯¯¯¯¯

--- Descriptives of each row-variable by groups of 'inclusion' ---

-------------------

row-variable: **gender**

0 1 0% 1% p.overall

[ALL] 9328 6185 60.13021 39.86979

0 5267 3217 62.08157 37.91843 0

1 4061 2968 57.77493 42.22507

-------------------

row-variable: **wc**

N mean sd lower upper p.overall

[ALL] 13776 102.6388 16.27914 102.3669 102.9107

0 7547 103.1944 17.23179 102.8056 103.5832 8e-06

1 6229 101.9656 15.01817 101.5926 102.3387

-------------------

row-variable: **sysRR**

N mean sd lower upper p.overall

[ALL] 14545 129.5266 18.08081 129.2327 129.8204

0 7944 129.6237 18.10081 129.2256 130.0218 0.476996

1 6601 129.4096 18.05739 128.9739 129.8453

-------------------

row-variable: **diaRR**

N mean sd lower upper p.overall

[ALL] 14528 83.12954 11.42153 82.9438 83.31528

0 7932 82.45058 11.49509 82.19757 82.70359 0

1 6596 83.94603 11.27931 83.67378 84.21828

-------------------

row-variable: **antihyp**

0 1 0% 1% p.overall

[ALL] 12343 2637 82.39653 17.60347

0 6105 2060 74.77036 25.22964 0

1 6238 577 91.53338 8.466618

-------------------

row-variable: **HDL**

N mean sd lower upper p.overall

[ALL] 8865 1.285778 0.497474 1.275421 1.296135

0 4639 1.233343 0.482695 1.21945 1.247237 0

1 4226 1.343336 0.507059 1.328044 1.358629

-------------------

row-variable: **statine**

0 1 0% 1% p.overall

[ALL] 12433 2547 82.99733 17.00267

0 6332 1833 77.55052 22.44948 0

1 6101 714 89.52311 10.47689

-------------------

row-variable: **triglyc**

N mean sd lower upper p.overall

[ALL] 8831 1.830691 1.164208 1.806406 1.854975

0 4614 1.896688 1.228918 1.86122 1.932157 0

1 4217 1.75848 1.084564 1.725736 1.791224

-------------------

row-variable: **fibr**

0 1 0% 1% p.overall

[ALL] 12433 2547 82.99733 17.00267

0 6332 1833 77.55052 22.44948 0

1 6101 714 89.52311 10.47689

-------------------

row-variable: **gluc**

N mean sd lower upper p.overall

[ALL] 8753 6.059822 1.904913 6.01991 6.099734

0 4578 6.398233 2.367989 6.32962 6.466846 0

1 4175 5.688745 1.093849 5.655555 5.721935

-------------------

row-variable: **diabmed**

0 1 0% 1% p.overall

[ALL] 13205 1775 88.15087 11.84913

0 6444 1721 78.92223 21.07777 0

1 6761 54 99.20763 0.79237

-------------------

row-variable: **MetS.crit**

0 1 2 3 4 5 0% 1% 2% 3% 4% 5% p.overall

[ALL] 2503 3649 3508 2164 1949 1404 16.49206 24.04296 23.11392 14.25842 12.8418 9.25084

0 1417 1792 1636 1102 1157 1174 17.11766 21.64774 19.76323 13.31239 13.97681 14.18217 0

1 1086 1857 1872 1062 792 230 15.74141 26.91694 27.13437 15.39354 11.47992 3.333816

-------------------

row-variable: MetS.prof

BGH BGHT BGHTW BGHW BGT BGTW BGW BHT BHTW BHW BTW GHT GHTW GHW GTW HTW BGH% BGHT% BGHTW% BGHW% BGT% BGTW% BGW% BHT%

[ALL] 33 262 1404 129 66 198 433 382 1065 246 334 152 295 59 53 406 0.598151 4.748958 25.44861 2.338227 1.196302 3.588907 7.848468 6.924053

0 17 216 1174 67 29 99 296 207 559 111 124 102 216 31 37 148 0.495194 6.291873 34.19749 1.951646 0.844742 2.883775 8.622196 6.029712

1 16 46 230 62 37 99 137 175 506 135 210 50 79 28 16 258 0.767754 2.207294 11.03647 2.975048 1.775432 4.75048 6.573896 8.397313

BHTW% BHW% BTW% GHT% GHTW% GHW% GTW% HTW% p.overall

[ALL] 19.30397 4.458945 6.054015 2.755121 5.347109 1.069422 0.960667 7.359072

0 16.28313 3.233324 3.612001 2.971162 6.291873 0.903 1.077775 4.311098 0

1 24.28023 6.477927 10.07678 2.399232 3.790787 1.34357 0.767754 12.38004

-------------------

row-variable: **age**

N mean sd lower upper p.overall

[ALL] 15513 45.97396 12.26406 45.78095 46.16696

0 8484 41.4604 13.64828 41.16994 41.75086 0

1 7029 51.42182 7.270353 51.25183 51.59182

-------------------

row-variable: **totalchol**

N mean sd lower upper p.overall

[ALL] 8910 4.945107 1.146373 4.9213 4.968913

0 4668 4.7281 1.130021 4.695675 4.760525 0

1 4242 5.183906 1.1165 5.150298 5.217514

-------------------

row-variable: **cursmoker**

0 1 0% 1% p.overall

[ALL] 6604 8909 42.57075 57.42925

0 3538 4946 41.70203 58.29797 0.016948

1 3066 3963 43.61929 56.38071

-------------------

row-variable: **DM**

0 1 0% 1% p.overall

[ALL] 13214 2299 85.18017 14.81983

0 6185 2299 72.90193 27.09807 0

1 7029 0 100 0

-------------------

row-variable: **histCVD**

0 1 0% 1% p.overall

[ALL] 12796 2717 82.48566 17.51434

0 5767 2717 67.97501 32.02499 0

1 7029 0 100 0 .

-------------------

row-variable: **Framingham**

N mean sd lower upper p.overall

[ALL] 8313 12.64444 12.47245 12.37629 12.9126

0 4355 12.73201 14.54735 12.29983 13.16418 0.494055

1 3958 12.54809 9.689974 12.24612 12.85007

-------------------

row-variable: **SCORE**

N mean sd lower upper p.overall

[ALL] 8521 1.527292 2.572937 1.472654 1.58193

0 4465 1.316605 2.818335 1.233916 1.399294 0

1 4056 1.759224 2.250002 1.68996 1.828489

-------------------

row-variable: **modifSCORE**

N mean sd lower upper p.overall

[ALL] 6087 9.56131 10.30507 9.302379 9.82024

0 2031 11.37957 11.7534 10.8681 11.89103 0

1 4056 8.650837 9.367072 8.362478 8.939195

-------------------

row-variable: **weight**

N mean sd lower upper p.overall

[ALL] 14768 89.12115 20.27035 88.7942 89.4481

0 8059 90.64058 20.94098 90.18331 91.09785 0

1 6709 87.29597 19.27805 86.83459 87.75735

-------------------

row-variable: **height**

N mean sd lower upper p.overall

[ALL] 14869 176.7271 10.04723 176.5656 176.8886

0 8106 176.8978 10.27595 176.6741 177.1215 0.022648

1 6763 176.5224 9.762848 176.2897 176.7551

-------------------

row-variable: **exsmoker**

0 1 0% 1% p.overall

[ALL] 12510 2762 81.91461 18.08539

0 6849 1429 82.73738 17.26262 0.004333

1 5661 1333 80.94081 19.05919

-------------------

row-variable: **antidep**

0 1 0% 1% p.overall

[ALL] 9835 5145 65.65421 34.34579

0 5430 2735 66.50337 33.49663 0.017389

1 4405 2410 64.63683 35.36317

-------------------

row-variable: **alcohol**

0 1 0% 1% p.overall

[ALL] 9170 6193 59.68886 40.31114

0 4985 3405 59.41597 40.58403 0.459384

1 4185 2788 60.01721 39.98279

-------------------

row-variable: **bipol**

0 1 0% 1% p.overall

[ALL] 12638 1151 91.65277 8.347233

0 6844 540 92.68689 7.313109 3e-06

1 5794 611 90.46058 9.539422

-------------------

row-variable: **othpsychosis**

0 1 0% 1% p.overall

[ALL] 4975 8814 36.07948 63.92052

0 2595 4789 35.14355 64.85645 0.01471

1 2380 4025 37.15847 62.84153

-------------------

row-variable: **unspecSMI**

0 1 0% 1% p.overall

[ALL] 9965 3824 72.26775 27.73225

0 5329 2055 72.16956 27.83044 0.79681

1 4636 1769 72.38095 27.61905

-------------------

row-variable: **secgenAP**

0 1 0% 1% p.overall

[ALL] 7266 8247 46.83814 53.16186

0 3933 4551 46.35785 53.64215 0.193298

1 3333 3696 47.41784 52.58216

-------------------

row-variable: **firstgenAP**

0 1 0% 1% p.overall

[ALL] 12546 2967 80.87411 19.12589

0 6943 1541 81.8364 18.1636 0.000876

1 5603 1426 79.71262 20.28738

-------------------

row-variable: **PRIMROSEdesk**

N mean sd lower upper p.overall

[ALL] 12299 4.506575 5.091583 4.416582 4.596568

0 6504 4.72124 6.40758 4.565489 4.876992 0

1 5795 4.265646 2.97225 4.189104 4.342187

-------------------

row-variable: **PRIMROSElipid**

N mean sd lower upper p.overall

[ALL] 7663 4.047637 4.501828 3.946826 4.148447

0 3950 4.239601 5.598698 4.064951 4.414252 8.8e-05

1 3713 3.843419 2.89908 3.750139 3.936699

**Missings**

--------Missingness table by 'inclusion'---------

_________________________________________________

0 1 p.overall

N=8484 N=7029

¯¯¯¯¯¯¯¯¯¯¯¯¯¯¯¯¯¯¯¯¯¯¯¯¯¯¯¯¯¯¯¯¯¯¯¯¯¯¯¯¯¯¯¯¯¯¯¯¯

gender 0 (0.00%) 0 (0.00%) .

wc 937 (11.0%) 800 (11.4%) 0.524

sysRR 540 (6.36%) 428 (6.09%) 0.500

diaRR 552 (6.51%) 433 (6.16%) 0.397

antihyp 319 (3.76%) 214 (3.04%) 0.017

HDL 3845 (45.3%) 2803 (39.9%) <0.001

statine 319 (3.76%) 214 (3.04%) 0.017

triglyc 3870 (45.6%) 2812 (40.0%) <0.001

fibr 319 (3.76%) 214 (3.04%) 0.017

gluc 3906 (46.0%) 2854 (40.6%) <0.001

diabmed 319 (3.76%) 214 (3.04%) 0.017

MetS.crit 206 (2.43%) 130 (1.85%) 0.016

MetS.prof 5051 (59.5%) 4945 (70.4%) <0.001

age 0 (0.00%) 0 (0.00%) .

totalchol 3816 (45.0%) 2787 (39.7%) <0.001

cursmoker 0 (0.00%) 0 (0.00%) .

DM 0 (0.00%) 0 (0.00%) .

histCVD 0 (0.00%) 0 (0.00%) .

Framingham 4129 (48.7%) 3071 (43.7%) <0.001

SCORE 4019 (47.4%) 2973 (42.3%) <0.001

modifSCORE 6453 (76.1%) 2973 (42.3%) 0.000

weight 425 (5.01%) 320 (4.55%) 0.198

height 378 (4.46%) 266 (3.78%) 0.041

exsmoker 206 (2.43%) 35 (0.50%) <0.001

antidep 319 (3.76%) 214 (3.04%) 0.017

alcohol 94 (1.11%) 56 (0.80%) 0.059

bipol 1100 (13.0%) 624 (8.88%) <0.001

othpsychosis 1100 (13.0%) 624 (8.88%) <0.001

unspecSMI 1100 (13.0%) 624 (8.88%) <0.001

secgenAP 0 (0.00%) 0 (0.00%) .

firstgenAP 0 (0.00%) 0 (0.00%) .

PRIMROSEdesk 1980 (23.3%) 1234 (17.6%) <0.001

PRIMROSElipid 4534 (53.4%) 3316 (47.2%) <0.001

2.MOPHAR

--------Summary descriptives table by 'inclusion'---------

________________________________________________

0 (excl.) 1 (included) p.overall

N=1202 N=748

¯¯¯¯¯¯¯¯¯¯¯¯¯¯¯¯¯¯¯¯¯¯¯¯¯¯¯¯¯¯¯¯¯¯¯¯¯¯¯¯¯¯¯¯¯¯¯¯

gender: 0.906

0 492 (40.9%) 309 (41.3%)

1 710 (59.1%) 439 (58.7%)

wc 98.7 (17.0) 100 (13.8) 0.050

sysRR 130 (19.3) 133 (18.1) <0.001

diaRR 80.5 (11.5) 84.0 (11.0) <0.001

antihyp: <0.001

0 967 (80.4%) 672 (89.8%)

1 235 (19.6%) 76 (10.2%)

HDL 1.34 (0.37) 1.44 (0.46) <0.001

statine: <0.001

0 1061 (88.3%) 721 (96.4%)

1 141 (11.7%) 27 (3.61%)

triglyc 1.42 (0.93) 1.52 (0.80) 0.059

fibr: 1.000

0 1201 (99.9%) 748 (100%)

1 1 (0.08%) 0 (0.00%)

gluc 5.69 (1.65) 5.55 (0.92) 0.100

diabmed: <0.001

0 1098 (91.3%) 743 (99.3%)

1 104 (8.65%) 5 (0.67%)

MetS.crit: <0.001

0 237 (19.7%) 90 (12.0%)

1 360 (30.0%) 250 (33.4%)

2 326 (27.1%) 248 (33.2%)

3 150 (12.5%) 95 (12.7%)

4 100 (8.32%) 51 (6.82%)

5 29 (2.41%) 14 (1.87%)

MetS.prof: .

BGH 14 (5.02%) 1 (0.62%)

BGHT 9 (3.23%) 2 (1.25%)

BGHTW 29 (10.4%) 14 (8.75%)

BGHW 37 (13.3%) 2 (1.25%)

BGT 1 (0.36%) 2 (1.25%)

BGTW 11 (3.94%) 7 (4.38%)

BGW 30 (10.8%) 18 (11.2%)

BHT 10 (3.58%) 4 (2.50%)

BHTW 37 (13.3%) 32 (20.0%)

BHW 55 (19.7%) 25 (15.6%)

BTW 23 (8.24%) 30 (18.8%)

GHTW 6 (2.15%) 8 (5.00%)

GHW 5 (1.79%) 4 (2.50%)

GTW 1 (0.36%) 0 (0.00%)

HTW 11 (3.94%) 11 (6.88%)

age 41.4 (18.5) 52.2 (7.95) <0.001

totalchol 4.66 (1.04) 5.35 (1.03) <0.001

cursmoker: 0.016

0 549 (58.5%) 399 (64.8%)

1 389 (41.5%) 217 (35.2%)

DM: <0.001

0 1039 (86.4%) 748 (100%)

1 163 (13.6%) 0 (0.00%)

histCVD: <0.001

0 896 (74.5%) 748 (100%)

1 306 (25.5%) 0 (0.00%)

Framingham 6.91 (11.9) 10.6 (8.54) <0.001

SCORE 1.14 (3.36) 1.38 (1.85) 0.176

modifSCORE 9.73 (8.31) 7.14 (8.04) 0.005

weight 82.6 (19.8) 83.0 (17.9) 0.604

height 174 (9.56) 174 (10.2) 0.360

exsmoker: 0.030

0 713 (76.0%) 437 (70.9%)

1 225 (24.0%) 179 (29.1%)

antidep: 0.021

0 724 (60.2%) 410 (54.8%)

1 478 (39.8%) 338 (45.2%)

alcohol: 0.097

0 598 (91.0%) 392 (94.0%)

1 59 (8.98%) 25 (6.00%)

bipol: <0.001

0 978 (86.9%) 561 (79.9%)

1 147 (13.1%) 141 (20.1%)

othpsychosis: 0.122

0 1088 (96.7%) 668 (95.2%)

1 37 (3.29%) 34 (4.84%)

unspecSMI: <0.001

0 184 (16.4%) 175 (24.9%)

1 941 (83.6%) 527 (75.1%)

secgenAP: <0.001

0 958 (79.7%) 540 (72.2%)

1 244 (20.3%) 208 (27.8%)

firstgenAP: 0.813

0 1173 (97.6%) 732 (97.9%)

1 29 (2.41%) 16 (2.14%)

PRIMROSEdesk 2.09 (4.12) 2.81 (2.17) <0.001

PRIMROSElipid 1.51 (3.16) 2.52 (2.18) <0.001

--- Descriptives of each row-variable by groups of 'inclusion' ---

-------------------

row-variable: **gender**

0 1 0% 1% p.overall

[ALL] 801 1149 41.07692 58.92308

0 492 710 40.93178 59.06822 0.906213

1 309 439 41.31016 58.68984

-------------------

row-variable: **wc**

N mean sd lower upper p.overall

[ALL] 1795 99.22451 15.88006 98.48939 99.95964

0 1095 98.66393 17.03632 97.65375 99.6741 0.050377

1 700 100.1014 13.84586 99.07395 101.1289

-------------------

row-variable: **sysRR**

N mean sd lower upper p.overall

[ALL] 1909 131.1446 18.90784 130.2959 131.9933

0 1176 129.9454 19.32846 128.8396 131.0512 0.000359

1 733 133.0685 18.05994 131.7589 134.3781

-------------------

row-variable: **diaRR**

N mean sd lower upper p.overall

[ALL] 1910 81.89162 11.42735 81.37882 82.40443

0 1177 80.54936 11.46207 79.89387 81.20486 0

1 733 84.04693 11.04252 83.24621 84.84765

-------------------

row-variable: **antihyp**

0 1 0% 1% p.overall

[ALL] 1639 311 84.05128 15.94872

0 967 235 80.44925 19.55075 0

1 672 76 89.83957 10.16043

-------------------

row-variable: **HDL**

N mean sd lower upper p.overall

[ALL] 1047 1.380984 0.409712 1.356138 1.40583

0 615 1.341317 0.369058 1.312092 1.370543 0.000305

1 432 1.437454 0.455977 1.394335 1.480573

-------------------

row-variable: **statine**

0 1 0% 1% p.overall

[ALL] 1782 168 91.38462 8.615385

0 1061 141 88.26955 11.73045 0

1 721 27 96.39037 3.609626

-------------------

row-variable: **triglyc**

N mean sd lower upper p.overall

[ALL] 1049 1.46326 0.880953 1.409888 1.516632

0 618 1.421392 0.929306 1.34798 1.494803 0.058594

1 431 1.523295 0.803836 1.447192 1.599398

-------------------

row-variable: **fibr**

0 1 0% 1% p.overall

[ALL] 1949 1 99.94872 0.051282

0 1201 1 99.91681 0.083195 1

1 748 0 100 0

-------------------

row-variable: **gluc**

N mean sd lower upper p.overall

[ALL] 1036 5.631081 1.396759 5.545928 5.716234

0 611 5.68527 1.649067 5.554253 5.816287 0.099715

1 425 5.553176 0.916307 5.465812 5.640541

-------------------

row-variable: **diabmed**

0 1 0% 1% p.overall

[ALL] 1841 109 94.41026 5.589744

0 1098 104 91.34775 8.652246 0

1 743 5 99.33155 0.668449

-------------------

row-variable: **MetS.crit**

0 1 2 3 4 5 0% 1% 2% 3% 4% 5% p.overall

[ALL] 327 610 574 245 151 43 16.76923 31.28205 29.4359 12.5641 7.74359 2.205128

0 237 360 326 150 100 29 19.71714 29.95008 27.12146 12.4792 8.319468 2.412646 0.000102

1 90 250 248 95 51 14 12.03209 33.42246 33.15508 12.70053 6.818182 1.871658

-------------------

row-variable: **MetS.prof**

BGH BGHT BGHTW BGHW BGT BGTW BGW BHT BHTW BHW BTW GHTW GHW GTW HTW BGH% BGHT% BGHTW% BGHW% BGT% BGTW%

[ALL] 15 11 43 39 3 18 48 14 69 80 53 14 9 1 22 3.416856 2.505695 9.794989 8.883827 0.683371 4.100228

0 14 9 29 37 1 11 30 10 37 55 23 6 5 1 11 5.017921 3.225806 10.39427 13.26165 0.358423 3.942652

1 1 2 14 2 2 7 18 4 32 25 30 8 4 0 11 0.625 1.25 8.75 1.25 1.25 4.375

BGW% BHT% BHTW% BHW% BTW% GHTW% GHW% GTW% HTW% p.overall

[ALL] 10.93394 3.189066 15.71754 18.22323 12.07289 3.189066 2.050114 0.22779 5.01139

0 10.75269 3.584229 13.26165 19.71326 8.243728 2.150538 1.792115 0.358423 3.942652 .

1 11.25 2.5 20 15.625 18.75 5 2.5 0 6.875

-------------------

row-variable: **age**

N mean sd lower upper p.overall

[ALL] 1950 45.53949 16.20638 44.81973 46.25925

0 1202 41.4193 18.51121 40.37177 42.46684 0

1 748 52.16043 7.94583 51.59008 52.73078

-------------------

row-variable: **totalchol**

N mean sd lower upper p.overall

[ALL] 1042 4.940787 1.089711 4.874545 5.007029

0 613 4.655465 1.036486 4.573252 4.737678 0

1 429 5.348485 1.033856 5.250376 5.446594

-------------------

row-variable: **cursmoker**

0 1 0% 1% p.overall

[ALL] 948 606 61.00386 38.99614

0 549 389 58.52878 41.47122 0.01572

1 399 217 64.77273 35.22727

-------------------

row-variable: **DM**

0 1 0% 1% p.overall

[ALL] 1787 163 91.64103 8.358974

0 1039 163 86.43927 13.56073 0

1 748 0 100 0

-------------------

row-variable: **histCVD**

0 1 0% 1% p.overall

[ALL] 1644 306 84.30769 15.69231

0 896 306 74.54243 25.45757 0

1 748 0 100 0

-------------------

row-variable: **Framingham**

N mean sd lower upper p.overall

[ALL] 904 8.488738 10.74321 7.787476 9.19

0 521 6.912825 11.87191 5.891035 7.934616 0

1 383 10.63247 8.542651 9.774212 11.49073

-------------------

row-variable: **SCORE**

N mean sd lower upper p.overall

[ALL] 888 1.239846 2.80835 1.054883 1.424809

0 504 1.136315 3.357684 0.842469 1.43016 0.176293

1 384 1.375731 1.851035 1.190005 1.561456

-------------------

row-variable: **modifSCORE**

N mean sd lower upper p.overall

[ALL] 489 7.693015 8.161615 6.967831 8.418199

0 105 9.73282 8.310695 8.124495 11.34114 0.00483

1 384 7.135256 8.041546 6.328399 7.942114

-------------------

row-variable: **weight**

N mean sd lower upper p.overall

[ALL] 1918 82.73806 19.11043 81.88227 83.59385

0 1180 82.56314 19.8165 81.43131 83.69496 0.604134

1 738 83.01775 17.93343 81.72177 84.31373

-------------------

row-variable: **height**

N mean sd lower upper p.overall

[ALL] 1920 174.0145 9.810565 173.5754 174.4536

0 1180 173.8498 9.564427 173.3036 174.3961 0.360246

1 740 174.277 10.19183 173.5415 175.0126

-------------------

row-variable: **exsmoker**

0 1 0% 1% p.overall

[ALL] 1150 404 74.00257 25.99743

0 713 225 76.01279 23.98721 0.029984

1 437 179 70.94156 29.05844

-------------------

row-variable: **antidep**

0 1 0% 1% p.overall

[ALL] 1134 816 58.15385 41.84615

0 724 478 60.23295 39.76705 0.020774

1 410 338 54.81283 45.18717

-------------------

row-variable: **alcohol**

0 1 0% 1% p.overall

[ALL] 990 84 92.17877 7.821229

0 598 59 91.01979 8.980213 0.097117

1 392 25 94.0048 5.995204

-------------------

row-variable: **bipol**

0 1 0% 1% p.overall

[ALL] 1539 288 84.23645 15.76355

0 978 147 86.93333 13.06667 8.2e-05

1 561 141 79.91453 20.08547

-------------------

row-variable: **othpsychosis**

0 1 0% 1% p.overall

[ALL] 1756 71 96.11385 3.886152

0 1088 37 96.71111 3.288889 0.121677

1 668 34 95.1567 4.843305

-------------------

row-variable: **unspecSMI**

0 1 0% 1% p.overall

[ALL] 359 1468 19.6497 80.3503

0 184 941 16.35556 83.64444 1e-05

1 175 527 24.92877 75.07123

-------------------

row-variable: **secgenAP**

0 1 0% 1% p.overall

[ALL] 1498 452 76.82051 23.17949

0 958 244 79.7005 20.2995 0.000166

1 540 208 72.19251 27.80749

-------------------

row-variable: **firstgenAP**

0 1 0% 1% p.overall

[ALL] 1905 45 97.69231 2.307692

0 1173 29 97.58735 2.412646 0.813274

1 732 16 97.86096 2.139037

-------------------

row-variable: **PRIMROSEdesk**

N mean sd lower upper p.overall

[ALL] 980 2.375698 3.50669 2.155877 2.595519

0 596 2.093967 4.123081 1.762278 2.425656 0.000389

1 384 2.812968 2.169713 2.595268 3.030668

-------------------

row-variable: **PRIMROSElipid**

N mean sd lower upper p.overall

[ALL] 620 1.93305 2.83605 1.709376 2.156725

0 363 1.513971 3.15505 1.188317 1.839624 3e-06

1 257 2.52498 2.184335 2.256656 2.793304

**MISSINGS**

--------Missingness table by 'inclusion'---------

________________________________________________

0 1 p.overall

N=1202 N=748

¯¯¯¯¯¯¯¯¯¯¯¯¯¯¯¯¯¯¯¯¯¯¯¯¯¯¯¯¯¯¯¯¯¯¯¯¯¯¯¯¯¯¯¯¯¯¯¯

gender 0 (0.00%) 0 (0.00%) .

wc 107 (8.90%) 48 (6.42%) 0.059

sysRR 26 (2.16%) 15 (2.01%) 0.941

diaRR 25 (2.08%) 15 (2.01%) 1.000

antihyp 0 (0.00%) 0 (0.00%) .

HDL 587 (48.8%) 316 (42.2%) 0.005

statine 0 (0.00%) 0 (0.00%) .

triglyc 584 (48.6%) 317 (42.4%) 0.009

fibr 0 (0.00%) 0 (0.00%) .

gluc 591 (49.2%) 323 (43.2%) 0.011

diabmed 0 (0.00%) 0 (0.00%) .

MetS.crit 0 (0.00%) 0 (0.00%) .

MetS.prof 923 (76.8%) 588 (78.6%) 0.379

age 0 (0.00%) 0 (0.00%) .

totalchol 589 (49.0%) 319 (42.6%) 0.007

cursmoker 264 (22.0%) 132 (17.6%) 0.025

DM 0 (0.00%) 0 (0.00%) .

histCVD 0 (0.00%) 0 (0.00%) .

Framingham 681 (56.7%) 365 (48.8%) 0.001

SCORE 698 (58.1%) 364 (48.7%) <0.001

modifSCORE 1097 (91.3%) 364 (48.7%) <0.001

weight 22 (1.83%) 10 (1.34%) 0.515

height 22 (1.83%) 8 (1.07%) 0.255

exsmoker 264 (22.0%) 132 (17.6%) 0.025

antidep 0 (0.00%) 0 (0.00%) .

alcohol 545 (45.3%) 331 (44.3%) 0.672

bipol 77 (6.41%) 46 (6.15%) 0.896

othpsychosis 77 (6.41%) 46 (6.15%) 0.896

unspecSMI 77 (6.41%) 46 (6.15%) 0.896

secgenAP 0 (0.00%) 0 (0.00%) .

firstgenAP 0 (0.00%) 0 (0.00%) .

PRIMROSEdeskb 606 (50.4%) 364 (48.7%) 0.480

PRIMROSEdeskw 606 (50.4%) 364 (48.7%) 0.480

PRIMROSEdesk 606 (50.4%) 364 (48.7%) 0.480

PRIMROSElipb 839 (69.8%) 491 (65.6%) 0.062

PRIMROSElipw 839 (69.8%) 491 (65.6%) 0.062

PRIMROSElipid 839 (69.8%) 491 (65.6%) 0.062

calyear 0 (0.00%) 0 (0.00%) .

¯¯¯¯¯¯¯¯¯¯¯¯¯¯¯¯¯¯¯¯¯¯¯¯¯¯¯

**II. Pairwise comparisons of continuous outcomes of CVD-risk models**



**Figure 3A**. Pairwise comparisons of CVD risk models, expressed in the number of MetS criteria fulfilled (PHAMOUS database). Lin's concordance correlation coefficients were from left to right (top): rho = 0.85 (0.84-0.86), rho = 0.25 (0.24-0.26), rho = 0.32 (0.31-0.33), and bottom: rho = 0.23 (0.22-0.24), rho = 0.37 (0.36-0.38), rho = 0.77 (0.76-0.78).



**Figure 3B**. Pairwise comparisons of CVD risk models, expressed in the number of MetS criteria fulfilled (MOPHAR database). Lin's concordance correlation coefficients were from left to right (top): rho = 0.91 (0.89-0.93), rho = 0.21 (0.18-0.25), rho = 0.30 (0.26-0.34), and bottom: rho = 0.20 (0.17-0.23), rho = 0.33 (0.29-0.37), rho = 0.78 (0.74-0.81).


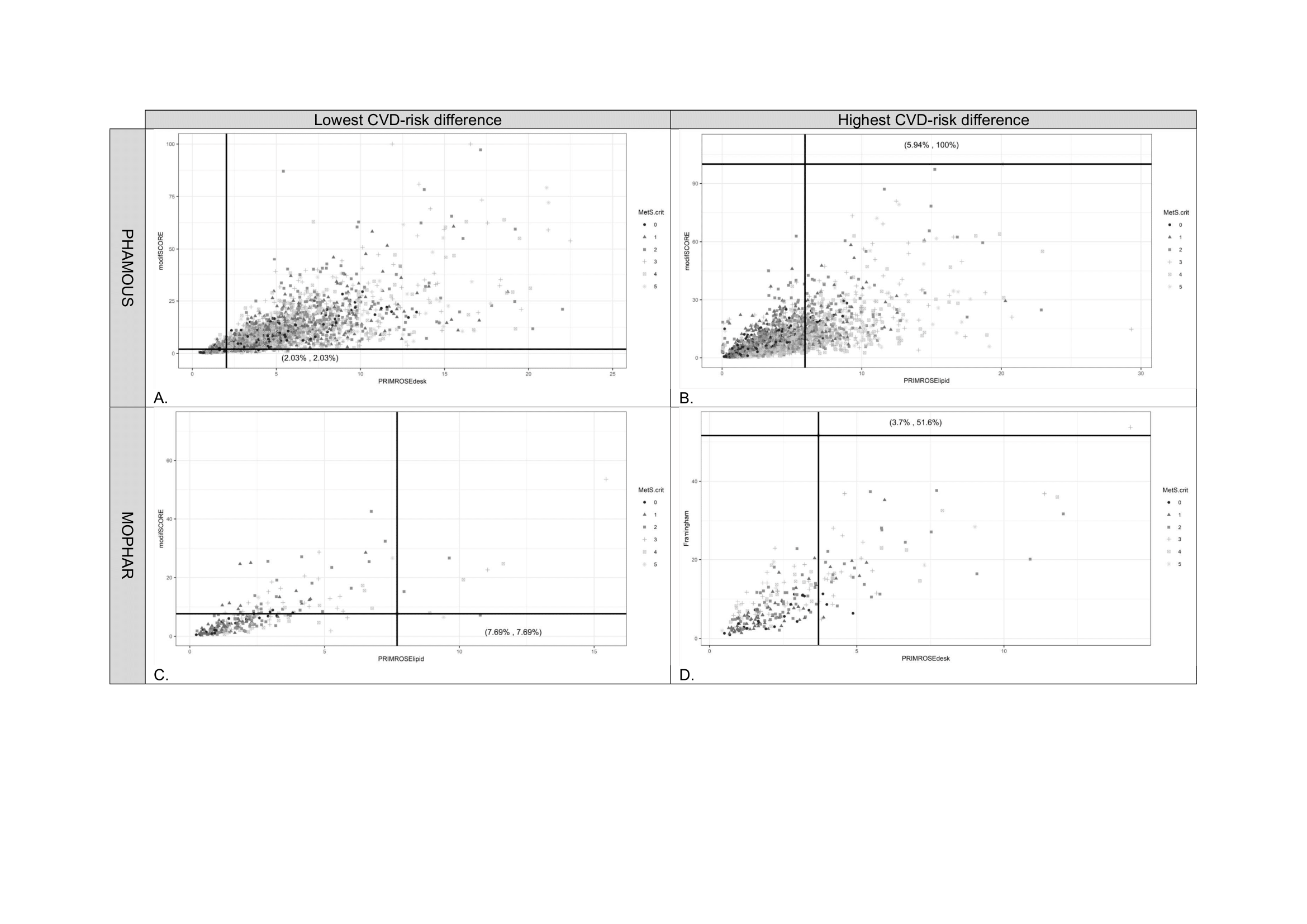
**Figure 4**. Lowest, respectively highest pairwise risk-differences from the various continuous CVR-assessment models applied to the same patient (i.e. intersection of the horizontal and vertical lines) in the PHAMOUS (top), and MOPHAR-database (bottom). The individual somatic parameters are specified here below.

|  | Pt. panel 4A | Pt. panel 4B | Pt. panel 4C | Pt. panel 4D |
| --- | --- | --- | --- | --- |
| Gender | Male | Male | Female | Male |
| Waist circumference (cm) | 113 | 113 | 89 | 118 |
| Systolic blood p. (mmHg) | 140 | 185 | 125 | 166 |
| Diastolic blood p. (mmHg) | 84 | 91 | 86 | 93 |
| Antihypertens. treat | No | No | Yes | No |
| HDL (mmol/L) | 1.24 | 2.5 | 1.10 | 0.50 |
| Statine treat. | Yes | No | No | No |
| Fibrates treat. | Yes | No | No | No |
| Triglycerides (mmol/L) | 1.59 | 1.70 | 1.99 | Missing |
| Glucose (mmol/L) | 4.9 | 5.7 | 4.9 | 12.9 |
| Hyperglycem. treat. | No | No | No | No |
| Age (years) | 42 | 63 | 58 | 46 |
| Total cholesterol (mmol/L) | 3.50 | 7.7 | 5.0 | 7.1 |
| Current smoker | No | Yes | Yes | Yes |
| Ex smoker | Yes | No | No | No |
| Weight (kg) | 96.6 | 100.30 | 62 | 98.9 |
| Height (cm) | 182 | 189 | 167 | 185 |
| Antidepressant treat. | No | No | Yes | Yes |
| 2nd generation antipsychotics | Yes | No | No | Yes |
| 1st generation antipsychotics | No | No | No | No |
| Excessive alcohol | Yes | Yes | No | No |
| Bipolar disorder | No | No | No | No |
| Other psychotic disorder | Yes | No | No | No |
| Unspecified SMI | No | Yes | Yes | Yes |

| # MetS criteria (profile) | 4 (BHTW) | 3 (BTW) | 4 (BHTW) | 4 (BGHW) |
| --- | --- | --- | --- | --- |

| Framingham | 3.9% | 43.59% | 14.6% | **51.62%** |
| --- | --- | --- | --- | --- |
| SCORE | 0.3% | 25.91% | 1.5% | 3.9% |
| Modified SCORE | **2.03%** | **100%** | **7.69%** | 17.31% |
| PRIMROSE desk | **2.03%** | 11.89% | 7.15% | **3.7%** |
| PRIMROSE lipid | 1.43% | **5.94%** | **7.69%** | 6.4% |

**Appendix 1**

1. **Metabolic syndrome diagnosis (based on ATP III criteria from NCEP)**

At least 3 of the following 5 (gender-specific) criteria had to be fulfilled.


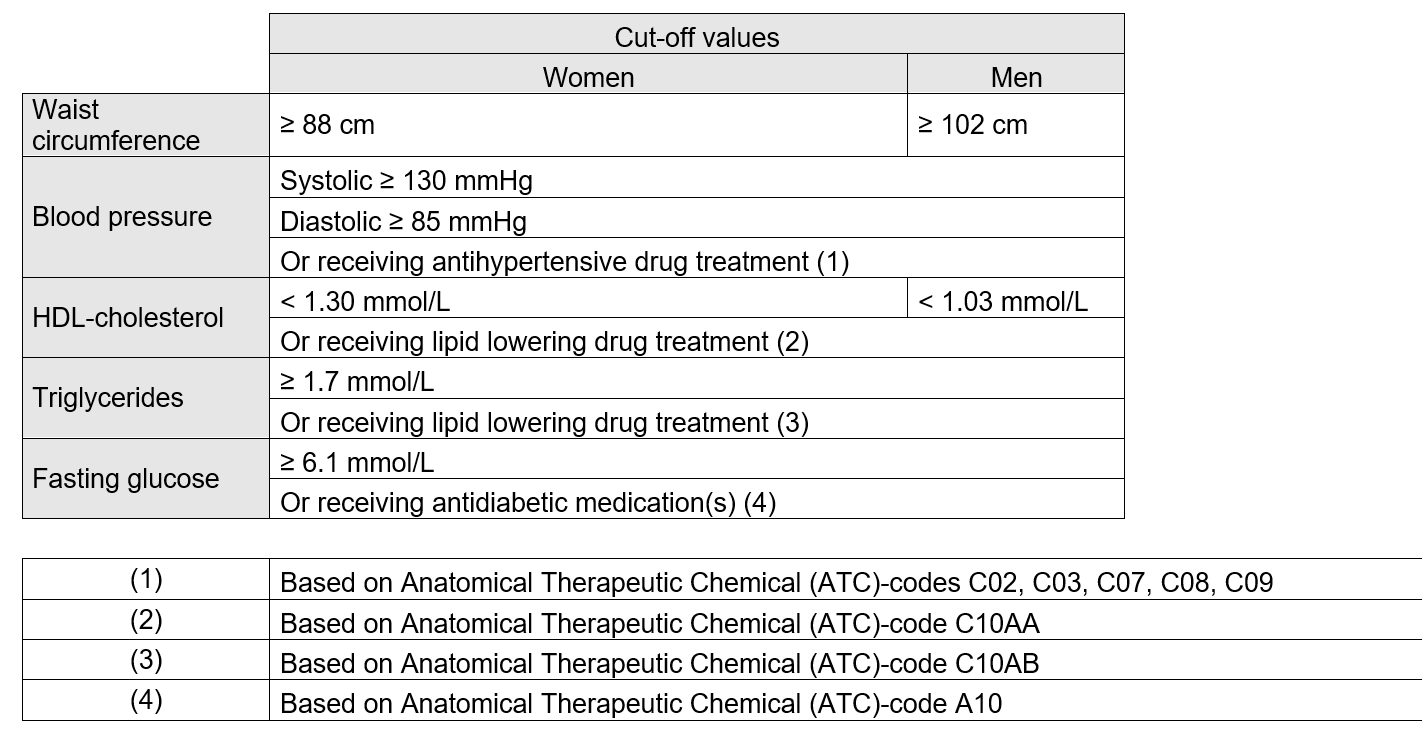


1. **Framingham risk score formula**

• The 10-year CVD-morbidity risk for **women** was calculated by the following Framingham formula. The total and HDL cholesterol were expressed in mg/dL (we converted our laboratory results, which were expressed in mmol/L, to mg/dL by multiplication with factor 38.6698). Systolic blood pressure was expressed in mmHg.

Risk factors = (ln(Age) * 2.32888) + (ln(Total cholesterol) * 1.20904) - (ln(HDL cholesterol) * 0.70833) + (ln(Systolic BP) * Hypertension medication factor) + Cig + DM - 26.1931

Risk = 100 * (1 - 0.95012^{e^Risk factors^})

The hypertension medication factor was 2.76157 for no medication, and 2.82263 for medication. The factor for cigarette smoker and diabetes were 0 if these were absent, and 0.52873, respectively 0.69154, if they were present.

• The 10-year CVD-morbidity risk for **men** was calculated by the following Framingham formula. The total and HDL cholesterol were expressed in mg/dL (we converted our laboratory results, which were expressed in mmol/L, to mg/dL by multiplication with factor 38.6698). Systolic blood pressure was expressed in mmHg.

Risk factors = (ln(Age) * 3.06117) + (ln(Total cholesterol) * 1.12370) - (ln(HDL cholesterol) * 0.93263) + (ln(Systolic BP) * Hypertension medication factor) + Cig + DM - 23.9802

Risk = 100 * (1 - 0.88936^{e^Risk factors^})

The hypertension medication factor was 1.93303 for no medication, and 1.99881 for medication. The factor for cigarette smoker and diabetes were 0 if these were absent, and 0.65451, respectively 0.57367, if they were present.

1. **SCORE risk score formula**

The SCORE estimates the 10-year CVD-mortality risk in 6 steps, with region and gender specific parameters. The cholesterol level is expressed in mmol/L and the systolic blood pressure in mmHg.

**Step 1**: Calculate the underlying survival probability S_0_ for coronary heart disease (CHD) and for non-coronary cardiovascular disease separately for the person’s age now and for their age in ten years time. The α and p values for the low risk region are shown below.

S_0_(age) = exp{-(exp(α))(age-20)^p^}
S_0_(age + 10) = exp{-(exp(α))(age-10)^p^}

CHD non-CHD CVD

α p α p
 __________________________________________________________

Low risk Men -22.1 4.71 -26.7 5.64

region Women -29.8 6.36 -31.0 6.62

CHD non-CHD CVD
 __________________________________________________________

Current smoker 0.71 0.63

Cholesterol (mmol/L) 0.24 0.02

Systolic BP (mmHg) 0.018 0.022

**Step 2**: Using the table above, calculate the weighted sums (for CHD and non-CHD CVD). Smoking is coded 1 for current and 0 for non-smoker.

w = ß_chol_(cholesterol – 6) + ß_SBP_(SBP – 120) + ß_smoker_(current)

**Step 3**: Combine the underlying risks for CHD and non-CHD CVD, at the person’s age and at their age ten years from now (four calculations) which were calculated at step 1 and 2, to get the probability of survival at each age for each cause.

S(age) = {S_0_(age)}^exp(w)^
S(age + 10) = {S_0_(age + 10)}^exp(w)^

**Step 4**: For each cause, calculate the 10-year survival probability based on the survival probability for the person’s current age and their age in 10 years time:

S_10_(age) = S(age + 10)/S(age)

**Step 5**: Calculate the 10 year risk for each end-point as

Risk_10_ = 1 – S_10_(age)

**Step 6**: Combine the risks for CHD and non-CHD CVD by adding them:

CVD Risk_10_(age) = [CHD Risk(age)] + [Non-CHD Risk(age)]

1. **PRIMROSE risk score formula**

There are two PRIMROSE models (i.e. the Lipid and the Desk model) that predict the 10-year CVD-morbidity risk. The systolic blood pressure is expressed in mmHg, total and HDL cholesterol in mmol/L, weight in kg, height in cm, deprivation is expressed in Townsend quintiles (1 = least deprived to 5 = most deprived).

The formulae use the following symbols.

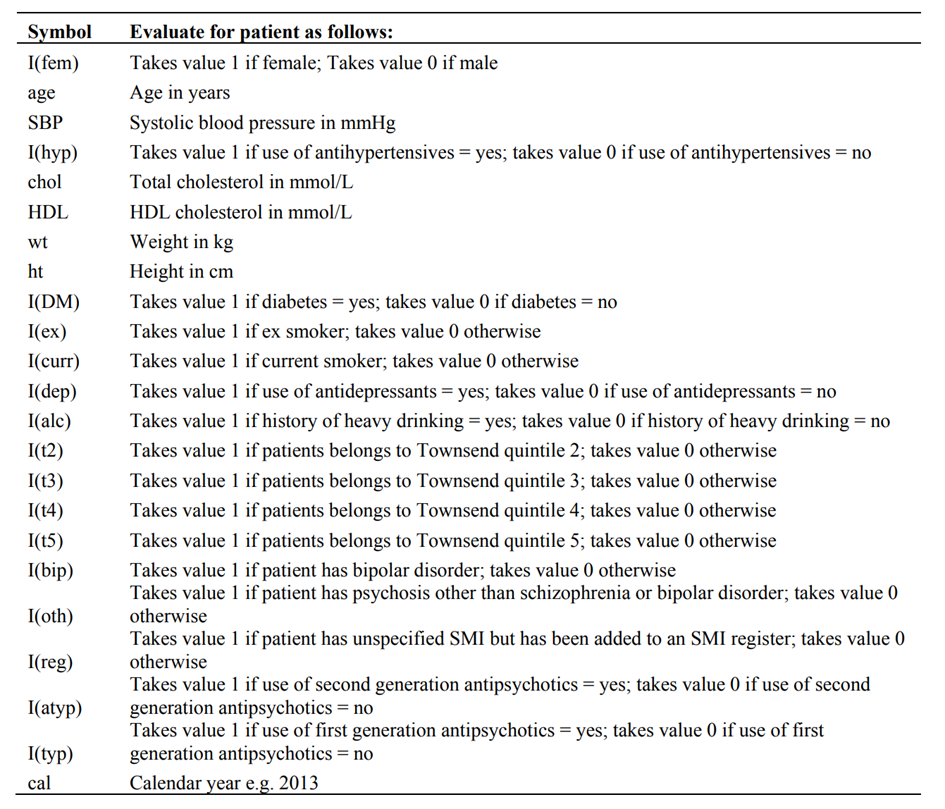


**Formula for PRIMROSE lipid model:**

Define P = - 0.1795×I(fem) + 3.78124×(ln(age) – 3.853361) + 0.007651×(SBP – 129.8673) + 0.625719×I(hyp) - 0.00796×I(hyp)×(SBP – 129.8673) + 0.11763×(chol – 5.562413) - 0.8183×(HDL – 1.389071) + 0.37734×I(DM) + 0.01639×I(ex) + 0.29659×I(curr) - 0.07043×(cal -2001.83) + 0.2104×I(dep) + 0.41392×I(alc) + 0.10963×I(t2) + 0.16388×I(t3) + 0.1828×I(t4) + 0.22126×I(t5) + 0.11177×I(bip) + 0.21004×I(oth) + 0.01526×I(reg) + 0.12121×I(atyp)

Define S = 0.968011

Then predicted risk (as a percentage) = 100×[1-S^exp(P)^]

**Formula for PRIMROSE desk model:**

Define P = - 0.49376×I(fem) + 3.50943×(ln(age) – 3.853361) + 0.00893×(SBP – 129.8673) + 0.65817264×I(hyp) - 0.00888×I(hyp)×(SBP – 129.8673) + 0.000680×(wt – 76.20105) - 0.0124×(ht – 167.9494) + 0.44971×I(DM) + 0.0738×I(ex) + 0.38081×I(curr) - 0.07524×(cal -2001.83) + 0.21846×I(dep) + 0.30721×I(alc) + 0.10919×I(t2) + 0.18412×I(t3) + 0.20238×I(t4) + 0.24762×I(t5) + 0.0978×I(bip) + 0.19063×I(oth) - 0.01138×I(reg) + 0.17662×I(atyp) + 0.1205×I(typ)

Define S = 0.951285

Then predicted risk (as a percentage) = 100×[1-S^exp(P)^]

__________________

The R-scripts can be viewed/downloaded from <https://www.dropbox.com/s/581dzk4vq7xfg3h/all_scripts.R?dl=0>
