# Supplementary material for: Cardiovascular risk assessment methods yield unequal risk predictions: a large cross-sectional study in psychiatric secondary care outpatients
Source: BMC Psychiatry. 2023 Jul 24;23:536. doi: 10.1186/s12888-023-05022-1 (PMC10367364; doi:10.1186/s12888-023-05022-1)
Supplement: Supplementary file 2 — Additional file 2: Supplementary document 2. [Quadackers_suppl2.docx; contains the mean CVD-risks stratified per MetS-criterium and MetS-profile]. [file 12888_2023_5022_MOESM2_ESM.docx]

**Contents supplementary material 2**

I. Mean CVD-risks stratified per MetS-criterium and MetS-profile

1. PHAMOUS 2

2. MOPHAR 7

**I. Mean CVD-risks stratified per MetS-criterium and MetS-profile**

1. **PHAMOUS**


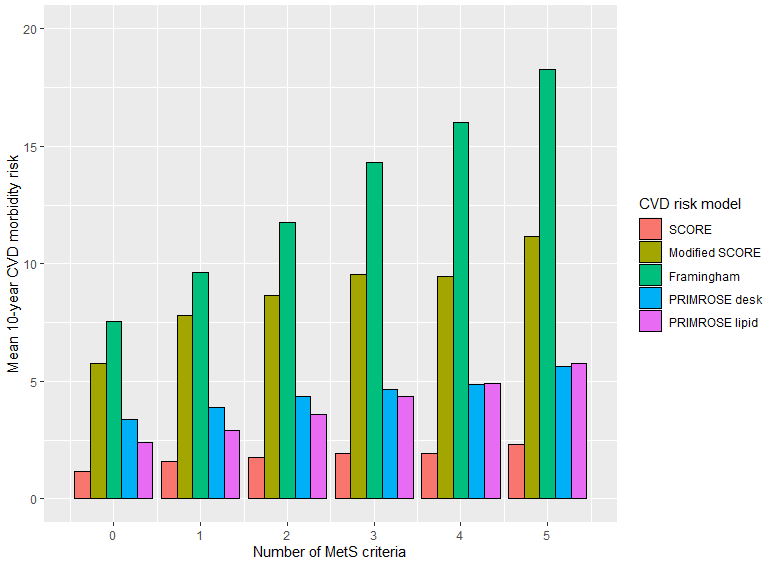


MetS.crit N **SCORE** sd se ci lower upper

1 0 424 1.172620 1.284240 0.06236820 0.1225902 1.05 1.30

2 1 842 1.581000 1.889738 0.06512466 0.1278259 1.45 1.71

3 2 976 1.753528 2.274200 0.07279536 0.1428536 1.61 1.90

4 3 923 1.949308 2.658211 0.08749608 0.1717146 1.78 2.12

5 4 663 1.916413 2.186810 0.08492867 0.1667620 1.75 2.08

6 5 228 2.306074 2.826514 0.18719045 0.3688531 1.94 2.67

7 NA 0 NaN NA NA NaN NaN NaN

MetS.crit N **modifSCORE** sd se ci lower upper

1 0 424 5.742503 5.226623 0.2538273 0.4989198 5.24 6.24

2 1 842 7.796827 7.995578 0.2755458 0.5408382 7.26 8.34

3 2 976 8.674538 9.769718 0.3127211 0.6136838 8.06 9.29

4 3 923 9.527987 10.594176 0.3487115 0.6843604 8.84 10.21

5 4 663 9.469466 9.197589 0.3572048 0.7013908 8.77 10.17

6 5 228 11.180295 11.707566 0.7753525 1.5278084 9.65 12.71

7 NA 0 NaN NA NA NaN NaN NaN

MetS.crit N **Framingham** sd se ci lower upper

1 0 408 7.552188 5.640637 0.2792531 0.5489585 7.00 8.10

2 1 815 9.648316 7.132017 0.2498236 0.4903745 9.16 10.14

3 2 955 11.771041 8.949068 0.2895850 0.5682973 11.20 12.34

4 3 897 14.313219 10.537264 0.3518290 0.6905049 13.62 15.00

5 4 656 15.993290 10.602504 0.4139582 0.8128453 15.18 16.81

6 5 227 18.276589 11.904612 0.7901369 1.5569775 16.72 19.83

7 NA 0 NaN NA NA NaN NaN NaN

MetS.crit N **PRIMROSEdesk** sd se ci lower upper

1 0 759 3.378923 2.241346 0.08135571 0.1597093 3.22 3.54

2 1 1535 3.878517 2.743342 0.07002060 0.1373462 3.74 4.02

3 2 1625 4.364229 2.958978 0.07340320 0.1439749 4.22 4.51

4 3 939 4.674770 3.141270 0.10251148 0.2011784 4.47 4.88

5 4 721 4.863141 3.214241 0.11970460 0.2350118 4.63 5.10

6 5 216 5.617982 3.898411 0.26525324 0.5228298 5.10 6.14

7 NA 0 NaN NA NA NaN NaN NaN

MetS.crit N PRIMROSElipid sd se ci lower upper

1 0 382 2.380604 1.648766 0.08435822 0.1658660 2.21 2.55

2 1 757 2.909812 2.194175 0.07974864 0.1565551 2.75 3.07

3 2 898 3.601603 2.601576 0.08681572 0.1703856 3.43 3.77

4 3 844 4.341747 3.080501 0.10603523 0.2081240 4.13 4.55

5 4 618 4.897197 3.195225 0.12853082 0.2524109 4.64 5.15

6 5 214 5.763337 3.714163 0.25389496 0.5004686 5.26 6.26

7 NA 0 NaN NA NA NaN NaN NaN


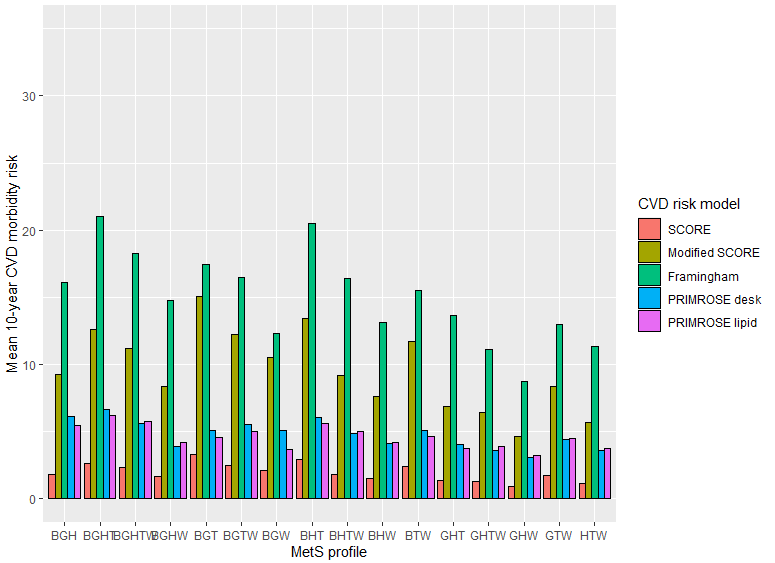


MetS.prof N **SCORE** sd se ci lower upper

1 BGH 16 1.7973121 1.365278 0.34131947 0.72750522 1.07 2.52

2 BGHT 44 2.6612969 2.640099 0.39800984 0.80266334 1.86 3.46

3 BGHTW 228 2.3060744 2.826514 0.18719045 0.36885307 1.94 2.67

4 BGHW 61 1.6803130 2.047713 0.26218274 0.52444356 1.16 2.20

5 BGT 37 3.2962884 4.159725 0.68385458 1.38692138 1.91 4.68

6 BGTW 98 2.5168791 2.750977 0.27789061 0.55153593 1.97 3.07

7 BGW 121 2.1113958 2.413353 0.21939572 0.43438826 1.68 2.55

8 BHT 126 2.9218041 4.017687 0.35792398 0.70837597 2.21 3.63

9 BHTW 384 1.8411336 2.089669 0.10663797 0.20966914 1.63 2.05

10 BHW 134 1.4879176 1.618306 0.13980037 0.27651971 1.21 1.76

11 BTW 210 2.3902754 3.070757 0.21190238 0.41774000 1.97 2.81

12 GHT 34 1.3436675 1.053604 0.18069154 0.36761971 0.98 1.71

13 GHTW 76 1.2807389 1.221486 0.14011408 0.27912157 1.00 1.56

14 GHW 28 0.9037516 0.944312 0.17845819 0.36616597 0.54 1.27

15 GTW 15 1.7294954 1.386428 0.35797422 0.76777833 0.96 2.50

16 HTW 202 1.1217542 1.220375 0.08586529 0.16931232 0.95 1.29

17 <NA> 2242 1.5788744 1.986499 0.04195372 0.08227221 1.50 1.66

MetS.prof N **modifSCORE** sd se ci lower upper

1 BGH 16 9.242324 5.761505 1.4403764 3.0700895 6.17 12.31

2 BGHT 44 12.588020 11.295450 1.7028531 3.4341306 9.15 16.02

3 BGHTW 228 11.180295 11.707566 0.7753525 1.5278084 9.65 12.71

4 BGHW 61 8.356536 8.548730 1.0945527 2.1894313 6.17 10.55

5 BGT 37 15.071407 16.955310 2.7874357 5.6531817 9.42 20.72

6 BGTW 98 12.257352 11.344486 1.1459662 2.2744256 9.98 14.53

7 BGW 121 10.507929 10.113413 0.9194012 1.8203504 8.69 12.33

8 BHT 126 13.427113 14.125355 1.2583866 2.4905033 10.94 15.92

9 BHTW 384 9.173646 8.789783 0.4485517 0.8819322 8.29 10.06

10 BHW 134 7.650809 7.030406 0.6073346 1.2012843 6.45 8.85

11 BTW 210 11.705222 12.678629 0.8749085 1.7247766 9.98 13.43

12 GHT 34 6.873224 4.638808 0.7955491 1.6185568 5.25 8.49

13 GHTW 76 6.457025 5.251591 0.6023988 1.2000399 5.26 7.66

14 GHW 28 4.649564 4.000926 0.7561040 1.5513973 3.10 6.20

15 GTW 15 8.339359 5.970437 1.5415601 3.3063177 5.03 11.65

16 HTW 202 5.709227 5.036348 0.3543562 0.6987325 5.01 6.41

17 <NA> 2242 7.790410 8.473973 0.1789655 0.3509555 7.44 8.14

MetS.prof N **Framingham** sd se ci lower upper

1 BGH 16 16.077415 9.092517 2.2731293 4.8450604 11.23 20.92

2 BGHT 43 21.031830 12.268400 1.8709135 3.7756563 17.26 24.81

3 BGHTW 227 18.276589 11.904612 0.7901369 1.5569775 16.72 19.83

4 BGHW 61 14.782987 10.337801 1.3236198 2.6476338 12.14 17.43

5 BGT 36 17.483729 12.755637 2.1259394 4.3158865 13.17 21.80

6 BGTW 97 16.507651 10.363595 1.0522636 2.0887268 14.42 18.60

7 BGW 118 12.351591 8.195260 0.7544349 1.4941187 10.86 13.85

8 BHT 123 20.531272 14.061878 1.2679169 2.5099681 18.02 23.04

9 BHTW 380 16.439554 10.668787 0.5472972 1.0761194 15.36 17.52

10 BHW 126 13.165731 8.982409 0.8002166 1.5837280 11.58 14.75

11 BTW 204 15.515192 10.488850 0.7343664 1.4479641 14.07 16.96

12 GHT 34 13.693264 8.332174 1.4289560 2.9072328 10.79 16.60

13 GHTW 75 11.162597 7.723760 0.8918629 1.7770757 9.39 12.94

14 GHW 28 8.774611 5.805612 1.0971575 2.2511812 6.52 11.03

15 GTW 13 13.009810 8.294398 2.3004520 5.0122544 8.00 18.02

16 HTW 199 11.382421 8.830312 0.6259642 1.2344124 10.15 12.62

17 <NA> 2178 10.186416 7.910302 0.1694978 0.3323944 9.85 10.52

MetS.prof N **PRIMROSEdesk** sd se ci lower upper

1 BGH 13 6.111238 4.118174 1.14217605 2.48858783 3.62 8.60

2 BGHT 40 6.647711 4.994810 0.78974887 1.59741787 5.05 8.25

3 BGHTW 216 5.617982 3.898411 0.26525324 0.52282982 5.10 6.14

4 BGHW 57 3.888348 2.758416 0.36536116 0.73190634 3.16 4.62

5 BGT 35 5.126003 3.399714 0.57465663 1.16784277 3.96 6.29

6 BGTW 89 5.525949 3.155822 0.33451645 0.66478114 4.86 6.19

7 BGW 123 5.122775 3.421069 0.30846740 0.61064202 4.51 5.73

8 BHT 150 6.100468 3.858917 0.31507929 0.62260084 5.48 6.72

9 BHTW 462 4.899269 3.140811 0.14612375 0.28715117 4.61 5.19

10 BHW 117 4.097508 2.845334 0.26305122 0.52100608 3.58 4.62

11 BTW 198 5.094079 3.182094 0.22614157 0.44596905 4.65 5.54

12 GHT 31 4.088911 2.138840 0.38414706 0.78453297 3.30 4.87

13 GHTW 73 3.609707 1.970337 0.23061046 0.45971356 3.15 4.07

14 GHW 28 3.052503 1.855455 0.35064801 0.71947029 2.33 3.77

15 GTW 12 4.398769 2.334076 0.67378984 1.48300143 2.92 5.88

16 HTW 232 3.588508 2.072615 0.13607387 0.26810453 3.32 3.86

17 <NA> 3919 3.983158 2.771804 0.04427666 0.08680748 3.90 4.07

MetS.prof N **PRIMROSElipid** sd se ci lower upper

1 BGH 14 5.442551 3.268518 0.87354808 1.8871859 3.56 7.33

2 BGHT 41 6.180592 4.529802 0.70743619 1.4297819 4.75 7.61

3 BGHTW 214 5.763337 3.714163 0.25389496 0.5004686 5.26 6.26

4 BGHW 57 4.171978 2.884603 0.38207497 0.7653881 3.41 4.94

5 BGT 35 4.590785 3.269038 0.55256835 1.1229540 3.47 5.71

6 BGTW 88 5.002948 3.326900 0.35464872 0.7049027 4.30 5.71

7 BGW 113 3.716765 2.367611 0.22272608 0.4413032 3.28 4.16

8 BHT 116 5.641137 3.985085 0.37000583 0.7329103 4.91 6.37

9 BHTW 360 5.032724 3.125145 0.16470962 0.3239169 4.71 5.36

10 BHW 116 4.229191 2.996026 0.27817401 0.5510092 3.68 4.78

11 BTW 195 4.665455 3.123709 0.22369347 0.4411834 4.22 5.11

12 GHT 32 3.741990 1.676926 0.29644142 0.6045963 3.14 4.35

13 GHTW 72 3.933619 2.287205 0.26954967 0.5374668 3.40 4.47

14 GHW 28 3.220950 2.129581 0.40245304 0.8257654 2.40 4.05

15 GTW 12 4.531924 2.732068 0.78868018 1.7358734 2.80 6.27

16 HTW 183 3.762468 2.809687 0.20769801 0.4098056 3.35 4.17

17 <NA> 2037 3.115541 2.345105 0.05195971 0.1018997 3.01 3.22

1. **MOPHAR**


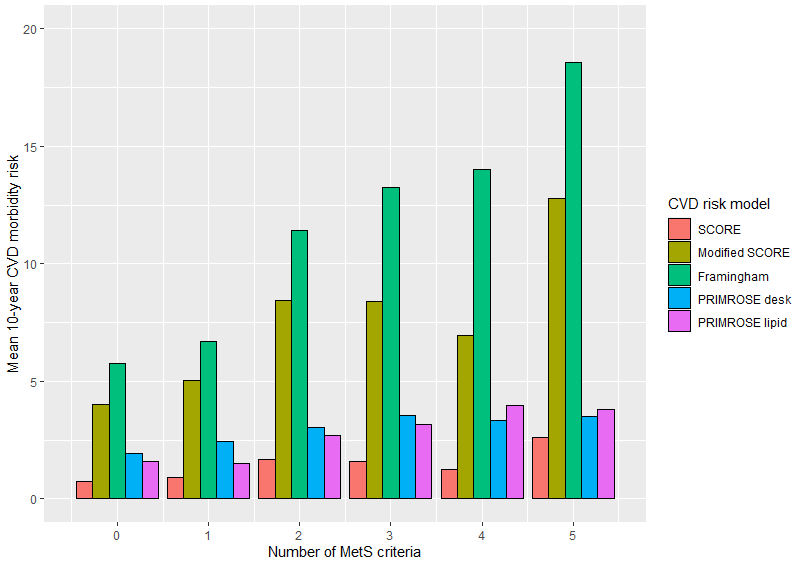


MetS.crit N **SCORE** sd se ci lower upper

1 0 35 0.7455460 0.6202559 0.1048424 0.2130653 0.53 0.96

2 1 96 0.9295724 1.2709557 0.1297164 0.2575196 0.67 1.19

3 2 119 1.6957672 2.3890701 0.2190057 0.4336909 1.26 2.13

4 3 75 1.6010538 1.7593805 0.2031558 0.4047967 1.20 2.01

5 4 47 1.2722995 1.3243293 0.1931733 0.3888377 0.88 1.66

6 5 12 2.6061773 2.9361405 0.8475908 1.8655347 0.74 4.47

MetS.crit N **modifSCORE** sd se ci lower upper

1 0 35 4.004619 2.657980 0.4492806 0.913048 3.09 4.92

2 1 96 5.041827 6.074378 0.6199636 1.230783 3.81 6.27

3 2 119 8.457258 9.940863 0.9112774 1.804577 6.65 10.26

4 3 75 8.400155 7.801042 0.9007868 1.794857 6.61 10.20

5 4 47 6.932936 5.682974 0.8289470 1.668584 5.26 8.60

6 5 12 12.790678 13.123359 3.7883875 8.338185 4.45 21.13

MetS.crit N **Framingham** sd se ci lower upper

1 0 35 5.766516 3.586842 0.6062869 1.232123 4.53 7.00

2 1 96 6.716818 5.954843 0.6077636 1.206563 5.51 7.92

3 2 118 11.432094 8.585814 0.7903883 1.565323 9.87 13.00

4 3 75 13.270688 8.410605 0.9711730 1.935104 11.34 15.21

5 4 47 14.009465 9.056623 1.3210442 2.659124 11.35 16.67

6 5 12 18.571758 14.295942 4.1268829 9.083208 9.49 27.65

MetS.crit N **PRIMROSEdesk** sd se ci lower upper

1 0 45 1.945199 1.215700 0.1812259 0.3652369 1.58 2.31

2 1 129 2.434497 1.746537 0.1537740 0.3042682 2.13 2.74

3 2 121 3.043882 2.326986 0.2115442 0.4188428 2.63 3.46

4 3 53 3.558812 2.618057 0.3596177 0.7216258 2.84 4.28

5 4 30 3.353607 2.445696 0.4465209 0.9132377 2.44 4.27

6 5 6 3.510121 3.685231 1.5044893 3.8674128 -0.36 7.38

MetS.crit N **PRIMROSElipid**  sd se ci lower upper

1 0 26 1.608315 1.006896 0.1974686 0.4066943 1.20 2.02

2 1 70 1.507273 1.134875 0.1356436 0.2706015 1.24 1.78

3 2 74 2.694458 2.032552 0.2362794 0.4709042 2.22 3.17

4 3 51 3.151245 2.532574 0.3546313 0.7122979 2.44 3.86

5 4 30 3.956977 2.802924 0.5117416 1.0466290 2.91 5.00

6 5 6 3.796975 3.726314 1.5212615 3.9105271 -0.11 7.71


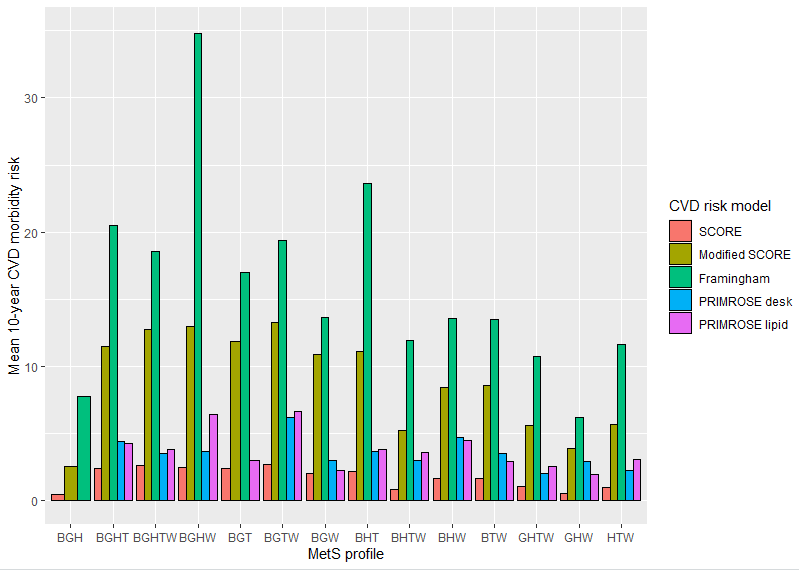


MetS.prof N **SCORE** sd se ci lower upper

1 BGH 1 0.4703945 NA NA NaN NaN NaN

2 BGHT 2 2.4136084 1.4838623 1.0492491 13.3319741 -10.92 15.75

3 BGHTW 12 2.6061773 2.9361405 0.8475908 1.8655347 0.74 4.47

4 BGHW 2 2.5071039 0.9877015 0.6984104 8.8741460 -6.37 11.38

5 BGT 2 2.4241323 0.8642758 0.6111353 7.7652097 -5.34 10.19

6 BGTW 6 2.7415138 2.1413658 0.8742089 2.2472256 0.49 4.99

7 BGW 12 2.0287395 1.1131317 0.3213334 0.7072501 1.32 2.74

8 BHT 2 2.1839717 0.4720130 0.3337636 4.2408689 -2.06 6.42

9 BHTW 29 0.8572685 0.7260072 0.1348162 0.2761584 0.58 1.13

10 BHW 17 1.6409623 2.8777746 0.6979629 1.4796152 0.16 3.12

11 BTW 27 1.6744701 1.5099974 0.2905991 0.5973351 1.08 2.27

12 GHTW 8 1.0808476 1.5080382 0.5331720 1.2607515 -0.18 2.34

13 GHW 3 0.5640479 0.3679021 0.2124084 0.9139196 -0.35 1.48

14 HTW 11 1.0225784 0.9907336 0.2987174 0.6655839 0.36 1.69

15 <NA> 250 1.2685174 1.8824532 0.1190568 0.2344867 1.03 1.50

MetS.prof N **modifSCORE** sd se ci lower upper

1 BGH 1 2.540130 NA NA NaN NaN NaN

2 BGHT 2 11.475199 5.809108 4.1076594 52.192761 -40.72 63.67

3 BGHTW 12 12.790678 13.123359 3.7883875 8.338185 4.45 21.13

4 BGHW 2 12.995753 6.100952 4.3140244 54.814877 -41.82 67.81

5 BGT 2 11.907784 3.763929 2.6614997 33.817560 -21.91 45.73

6 BGTW 6 13.313919 8.740081 3.5681231 9.172152 4.14 22.49

7 BGW 12 10.900110 5.665184 1.6353978 3.599486 7.30 14.50

8 BHT 2 11.138255 2.407266 1.7021945 21.628431 -10.49 32.77

9 BHTW 29 5.237105 3.523103 0.6542238 1.340117 3.90 6.58

10 BHW 17 8.463060 12.588150 3.0530749 6.472230 1.99 14.94

11 BTW 27 8.602536 6.472481 1.2456296 2.560428 6.04 11.16

12 GHTW 8 5.643312 5.724428 2.0238910 4.785742 0.86 10.43

13 GHW 3 3.886234 2.282276 1.3176727 5.669488 -1.78 9.56

14 HTW 11 5.707174 4.520305 1.3629231 3.036782 2.67 8.74

15 <NA> 250 6.522363 8.086923 0.5114619 1.007343 5.52 7.53

MetS.prof N **Framingham** sd se ci lower upper

1 BGH 1 7.802085 NA NA NaN NaN NaN

2 BGHT 2 20.529956 2.725923 1.9275189 24.4914492 -3.96 45.02

3 BGHTW 12 18.571758 14.295942 4.1268829 9.0832081 9.49 27.65

4 BGHW 2 34.793304 23.801938 16.8305119 213.8519296 -179.06 248.65

5 BGT 2 17.008496 1.562594 1.1049209 14.0393511 2.97 31.05

6 BGTW 6 19.357679 12.719973 5.1929073 13.3487932 6.01 32.71

7 BGW 12 13.694797 5.176102 1.4942120 3.2887383 10.41 16.98

8 BHT 2 23.663115 1.104595 0.7810665 9.9243912 13.74 33.59

9 BHTW 29 11.913028 5.158098 0.9578348 1.9620357 9.95 13.88

10 BHW 17 13.552843 12.235263 2.9674871 6.2907916 7.26 19.84

11 BTW 27 13.516294 7.641676 1.4706412 3.0229463 10.49 16.54

12 GHTW 8 10.771806 6.689077 2.3649459 5.5922084 5.18 16.36

13 GHW 3 6.192636 2.425315 1.4002560 6.0248152 0.17 12.22

14 HTW 11 11.627507 7.429678 2.2401322 4.9913257 6.64 16.62

15 <NA> 249 8.817790 7.509947 0.4759236 0.9373675 7.88 9.76

MetS.prof N **PRIMROSEdesk** sd se ci lower upper

1 BGH 0 NaN NA NA NaN NaN NaN

2 BGHT 2 4.396735 3.242530 2.2928150 29.1329769 -24.74 33.53

3 BGHTW 6 3.510121 3.685231 1.5044893 3.8674128 -0.36 7.38

4 BGHW 1 3.700412 NA NA NaN NaN NaN

5 BGT 0 NaN NA NA NaN NaN NaN

6 BGTW 4 6.185071 4.546992 2.2734962 7.2352797 -1.05 13.42

7 BGW 7 3.006009 1.293269 0.4888096 1.1960739 1.81 4.20

8 BHT 2 3.716588 2.112320 1.4936355 18.9784380 -15.26 22.70

9 BHTW 17 3.004201 1.723033 0.4178969 0.8859018 2.12 3.89

10 BHW 12 4.740509 3.756955 1.0845394 2.3870551 2.35 7.13

11 BTW 23 3.549336 2.521116 0.5256891 1.0902124 2.46 4.64

12 GHTW 6 2.050440 1.165361 0.4757565 1.2229710 0.83 3.27

13 GHW 2 2.930766 1.240418 0.8771081 11.1447152 -8.21 14.08

14 HTW 7 2.251345 1.504784 0.5687549 1.3916932 0.86 3.64

15 <NA> 295 2.609809 1.979403 0.1152453 0.2268103 2.38 2.84

MetS.prof N **PRIMROSElipid** sd se ci lower upper

1 BGH 0 NaN NA NA NaN NaN NaN

2 BGHT 2 4.306540 3.0759524 2.1750268 27.6363355 -23.33 31.94

3 BGHTW 6 3.796975 3.7263144 1.5212615 3.9105271 -0.11 7.71

4 BGHW 1 6.413313 NA NA NaN NaN NaN

5 BGT 1 2.996449 NA NA NaN NaN NaN

6 BGTW 4 6.680188 4.9922171 2.4961086 7.9437315 -1.26 14.62

7 BGW 7 2.259559 1.0505123 0.3970563 0.9715618 1.29 3.23

8 BHT 2 3.864230 1.8899846 1.3364209 16.9808377 -13.12 20.85

9 BHTW 17 3.611437 2.2855535 0.5543281 1.1751232 2.44 4.79

10 BHW 10 4.464974 4.3309460 1.3695654 3.0981721 1.37 7.56

11 BTW 22 2.907734 2.0385046 0.4346106 0.9038223 2.00 3.81

12 GHTW 6 2.594623 1.4778048 0.6033113 1.5508610 1.04 4.15

13 GHW 2 2.000742 0.3820048 0.2701182 3.4321770 -1.43 5.43

14 HTW 7 3.078615 2.0985621 0.7931819 1.9408463 1.14 5.02

15 <NA> 170 2.039501 1.6715561 0.1282025 0.2530846 1.79 2.29
